# Rewilding processes shape the use of Mediterranean landscapes by an avian top scavenger

**DOI:** 10.1038/s41598-020-59591-2

**Published:** 2020-02-18

**Authors:** P. Martin-Díaz, A. Cortés-Avizanda, D. Serrano, E. Arrondo, J. A. Sánchez-Zapata, J. A. Donázar

**Affiliations:** 1 Department of Conservation Biology, Estación Biológica de Doñana (CSIC), Seville, Spain; 2 Animal Ecology and Demography Unit, IMEDEA (CSIC-UIB), Balearic Islands (Mallorca), Spain; 3 Department of applied Biology, University Miguel Hernández, Elche (Alicante), Spain

**Keywords:** Biodiversity, Conservation biology, Population dynamics

## Abstract

The Mediterranean biome has seen a great decline in its rural population. This trend has been followed by an abandonment of agricultural and livestock practices, which has provided an opportunity for rewilding to take place. Rewilding processes can modify the availability of carrion resources for avian obligate scavengers and reduce accessible open areas due to the increase of shrub and forest. We examined how changes in landscape configuration in the past five decades (1956–2011) mediate the foraging behaviour of griffon vultures. Particularly, we examined whether vultures use those areas under natural succession and with a high availability of wild ungulate carcasses. We used GPS information yielded by 30 adult griffon vultures exploiting large regions of southern Spain. We determined (a) habitat use considering land uses and food availability and (b) how tracked individuals responded to areas in different stages of rewilding. Our results showed that vultures preferentially used Mediterranean scrublands, woodlands and the agroforest Mediterranean ecosystem called dehesa, as well as areas with high food resources, namely wild ungulates in winter and a mixture of wild ungulates and livestock in summer. Due to a higher abundance of wild ungulates, vultures forage preferentially in areas with low levels of rewilding, either for being in the first stages of natural succession or for not having experienced further rewilding since the middle of the last century. Rewilding processes are expected to continue in the future affecting the scavenger guild structure and function deeply. Improved management will be essential to preserve ecological processes, ecosystem services and populations of endangered species.

## Introduction

Millennia-old agricultural and livestock activities have almost entirely shaped Mediterranean landscapes^1^. Traditional low-intensive agro-grazing practices have led to a range of cultural landscapes^2,3^, that often harbour exceptional conservation value^4^. Human-mediated transformations devoted to extensive agro-grazing historically led to subtle and slow transformations that resulted in the current landscape heterogeneities, to which other organisms have adapted or from which they have even benefited^5^. However, the technological development and deep socio-economic transformations that have occurred in Europe since the middle of the last century have brought a new wave of major changes, including the intensification of high-yield agricultural areas and progressive abandonment of marginal lands^5^.

While landscape transformation, homogenisation, overgrazing and a loss of genetic diversity and biodiversity are well-known consequences of agricultural intensification^6,7^, less focus has been given to the consequences of traditional land use changes as well as the reduction of human control on landscapes^5,8^. Within this scenario, the Mediterranean biome has been undergoing an important decline in its rural population followed by an abandonment of agricultural land, pastures and traditional livestock practices (−15% between 1970–2010^5^), a trend that is foreseen to escalate in the future^9,10^. This exodus of people has been noted as a potential opportunity to favour the natural succession of vegetation towards scrubs and forests, so-called “ecological rewilding”^8,11^.

Ecological succession after abandonment allows the increase of biodiversity and favours interactions between species, and could ultimately restore key ecosystem processes^5^. Nevertheless, the impacts are nearly unknown for organisms involved in key ecosystem functional processes such as scavenging. Vultures, as obligate scavengers, play a prominent role in nutrient cycling and food chains by feeding on carcasses, while providing supporting and cultural ecosystem services^12,13^. Vultures are an iconic example of the global “biodiversity crisis”, given the abrupt decline suffered by many populations worldwide during the 20th century^14^. European vultures in particular have been heavily dependent on livestock carcasses associated with human activities during the last centuries^15,16^, which have made them highly vulnerable to sanitary policies concerned with the management of dead livestock^16^.

The generalised land abandonment and concomitant rewilding process predicted for the next few decades present a new scenario for vulture conservation^17^. If the regeneration of natural vegetation is accompanied by the recovery of wild ungulates, vultures could benefit from this resource, compensating for the shortage of domestic livestock. This could lead to changes in their foraging strategies and the way they exploit landscapes^17,18^. In this vein, because obligate scavengers have developed a high capacity to scan wide areas^19^, a plastic behavioural response to large-scale mosaic landscapes could be predicted. Vultures could need to adjust their foraging strategies in the future if rewilding scenarios affect food availability and/or change the spatial distribution of food resources. However, rewilding implies a densification of the vegetation, so vultures might have more difficulty in finding and accessing carcasses^20^.

Despite this, there is a general lack of knowledge about whether large vultures are able to adjust their foraging strategies to changes in the distribution and/or accessibility to trophic resources^21,22^, shaped by rewilding landscapes. To fill this gap, we used information from GPS-tracked griffon vultures (*Gyps fulvus*), obligate avian scavengers that provide important regulating ecosystem services in Mediterranean Europe^18,23^ (see above). Here, we aim to determine how changes in the past decades in landscape configuration have influenced current foraging strategies of adult griffon vultures. We first predicted that griffon vultures will preferentially use those habitats holding more trophic resources (wild and domestic ungulates) and that they will respond to seasonal fluctuations in their availability. Because rewilding implies an increase in carrion resources derived from wild ungulate expansion and, in parallel, less accessible foraging areas given the increase of shrub and forest, we secondly predict that foraging griffons will preferentially use those areas where both Mediterranean habitats are under natural succession and wild ungulates are present at higher densities.

## Results

**Figure 1:**
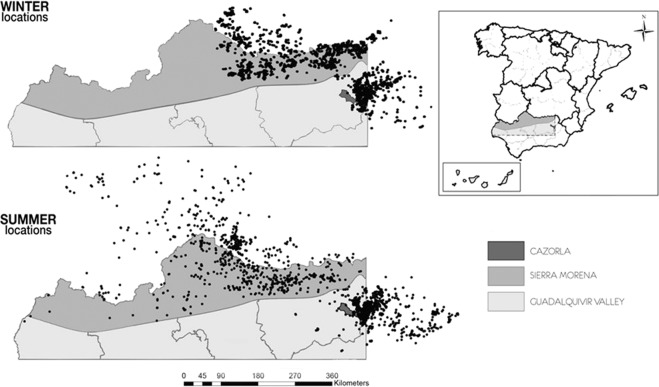
Study area and locations (dots) of foraging vultures in winter and summer. Different shading shows the Sierra Morena mountain area, the Guadalquivir Valley and the breeding area in Sierra de Cazorla.

The 30 GPS-marked griffon vultures yielded a total number of 117,157 locations in winter and 288,847 in summer. After the filtering process (see Methods), there were 23,102 and 20,044 locations, respectively. The population home range of the studied vultures (kernel 95%) was 10,253.85 km^2^ in winter and 99,883.23 km^2^ in summer. Individual home ranges ranged between 395.68 km^2^ and 4,261.05 km^2^ in winter (Mean = 2,128.17, SD = 1,000.83) and between 1,015.32 km^2^ and 15,814.44 km^2^ in summer (Mean = 6,897.46, SD = 3,966.52). Wintering locations were concentrated in the NE of the study area, relatively close to the breeding grounds. During summer, the vultures foraged in a much wider area, reaching the western ranges of Sierra Morena and the Portuguese border (Fig. 1).

### Foraging habitat use

The Compositional Analysis (Table 1) revealed that in winter, the ranking of preferred habitats was similar at both population (λ = 0.122, P = 0.002) and individual (λ = 0.108, P = 0.002) levels: scrubs followed by Mediterranean forest, dehesa and grassland. In summer, considering the population home range scale (λ = 0.044, P = 0.002), the ranking of preferred habitats was grassland, scrubs, Mediterranean forest and dehesa. At an individual home-range scale (λ = 0.247, P = 0.002), the order was scrubs, followed by Mediterranean forest, grassland and dehesas.

**Table 1:** Simplified ranking matrices resulting from the Compositional Analyses of habitat selection by foraging griffon vultures (n = 30).

Rank	Habitat	Dehesa	Farming	Grassland	Medit. forest	Others	Reforestation	Scrubs
**Winter**								
**Common**								
3	Dehesa	0	+++	+	−−−	+++	+++	−−−
7	Farming	−−−	0	−−−	−−−	−	−−−	−−−
4	Grassland	−	+++	0	−−−	+++	+	−−−
2	Medit. forest	+++	+++	+++	0	+++	+++	−
6	Others	−−−	+	−−−	−−−	0	−−−	−−−
5	Reforestation	−−−	+++	−	−−−	+++	0	−−−
1	Scrubs	+++	+++	+++	+	+++	+++	0
**Individual**								
3	Dehesa	0	+++	+	−	+++	+++	−−−
7	Farming	−−−	0	−−−	−−−	−	−−−	−−−
4	Grassland	−	+++	0	−−−	+++	+++	−−−
2	Medit. forest	+	+++	+++	0	+++	+++	−−−
6	Others	−−−	+	−−−	−−−	0	−−−	−−−
5	Reforestation	−−−	+++	−−−	−−−	+++	0	−−−
1	Scrubs	+++	+++	+++	+++	+++	+++	0
**Summer**								
**Common**								
4	Dehesa	0	+++	−−−	−−−	+++	+	−−−
7	Farming	−−−	0	−−−	−−−	−	−−−	−−−
1	Grassland	+++	+++	0	+++	+++	+++	+++
3	Medit. forest	+++	+++	−−−	0	+++	+++	−
6	Others	−−−	+	−−−	−−−	0	−	−−−
5	Reforestation	−	+++	−−−	−−−	+	0	−−−
2	Scrubs	+++	+++	−−−	+	+++	+++	0
4	Dehesa	0	+++	−−−	−−−	+++	+	−−−
**Individual**								
4	Dehesa	0	+++	−	−	+++	+++	−−−
7	Farming	−−−	0	−−−	−−−	−−−	−−−	−−−
3	Grassland	+	+++	0	−	+++	+++	−−−
2	Medit. forest	+	+++	+	0	+++	+++	−
5	Others	−−−	+++	−−−	−−−	0	+	−−−
6	Reforestation	−−−	+++	−−−	−−−	−	0	−−−
1	Scrubs	+++	+++	+++	+	+++	+++	0
4	Dehesa	0	+++	−	−	+++	+++	−−−

We distinguished two scales: i) *Common*: proportional habitat use within the kernel (95%) for all the birds as compared to the total availability of habitat types, and ii) *Individual*: proportions of locations for each bird in each habitat type as compared to the availability of each habitat type within the individual home range (kernel 95%). Each mean element in the matrix was replaced by its sign; a triple sign represents significant deviation from random at p < 0.05. We distinguished winter and summer seasons.

**Figure 2:**
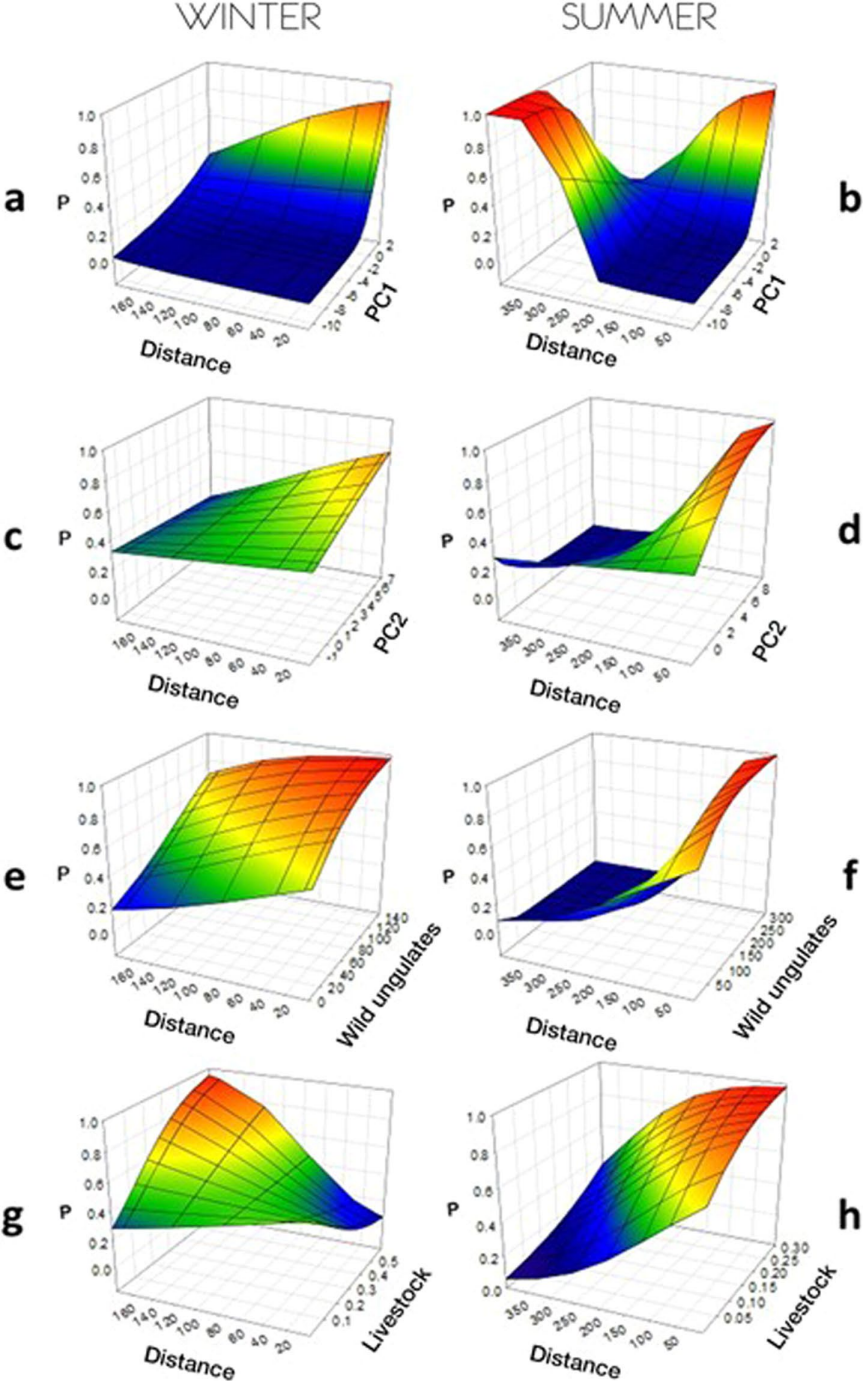
Graphical representation of the probability that GPS-marked griffon vultures visit 1 × 1 km grid-cells in relation to the trade-off between distance to the breeding area and habitat and food availability variables in the two study seasons. PC1: coordinates of the cell in Principal Component 1 describing a gradient from humanised (−) to natural habitats (+); PC2: gradient from natural (−) to reforested woodland (+); Wild ungulates: index of abundance obtained from hunting statistics; Livestock: index of abundance obtained from farm statistics. Note that the variables are shown at a different scale for each season.

GLMMs revealed that cells with natural habitats (positive values of PC1) and close to the breeding area were more likely visited by foraging griffon vultures in the two seasons (Table 2, Fig. 2a,b). Conversely, humanised areas (negative values in PC1) were only visited in summer and far from the breeding area (Fig. 2b). The effect of the transition between Mediterranean forests and reforested woods (from negative to positive values of PC2) was less marked in winter near the breeding area (Table 2, Fig. 2c). In distant areas, however, there was a slight trend for vultures to select dehesas and woodlands (Fig. 2d).

**Table 2:** Selected GLMMs (lowest AICc) describing habitat use by GPS-marked griffon vultures during winter and summer.

Effect	Estimate	S.E.	d.f.
**Winter**			
Intercept	−0.07387	0.05569	29
Distance	−0.5311	0.01516	46141
PC1	0.8821	0.01512	46141
Distance*PC1	−0.3551	0.01579	46141
PC2	−0.06861	0.01200	46141
Distance*PC2	0.09463	0.01282	46141
Wild ungulates	0.2582	0.01618	46141
Distance*Wild ungulates	0.04855	0.01217	46141
Livestock	−0.02184	0.02063	46141
Distance*Livestock	0.09648	0.01396	46141
**Summer**			
Intercept	0.06552	0.24670	26
Distance	−0.6265	0.01723	39832
PC1	1.5070	0.02094	39832
Distance*PC1	−0.6031	0.01902	39832
PC2	−0.1165	0.01486	39832
Distance*PC2	0.06809	0.01480	39832
Wild ungulates	−0.2017	0.01700	39832
Distance*Wild ungulates	−0.1405	0.01904	39832
Livestock	0.2106	0.01955	39832
Distance*Livestock	−0.06291	0.01259	39832

The relationship between the probability of vulture presence and wild ungulate and livestock densities were complex depending on distance to the colony (Table 2, Fig. 2e–h). In summer, the areas with wild ungulates were exclusively used when they were very close to the breeding area (Fig. 2f). During winter, the vultures used areas with high densities of wild ungulates, the probability of presence decreasing slowly with the distance to the breeding area (Fig. 2e). Regarding livestock, the probability of vulture presence was highest in cells with high livestock-density values far from the breeding area in winter, while they seem to avoid grid-cells with many livestock close to the colony (Table 2, Fig. 2g). During summer, birds were more likely to visit areas with greater livestock numbers, with their higher probabilities of occurrence at short and medium distances from the breeding area (Table 2, Fig. 2h).

### Habitat use and rewilding

**Figure 3:**
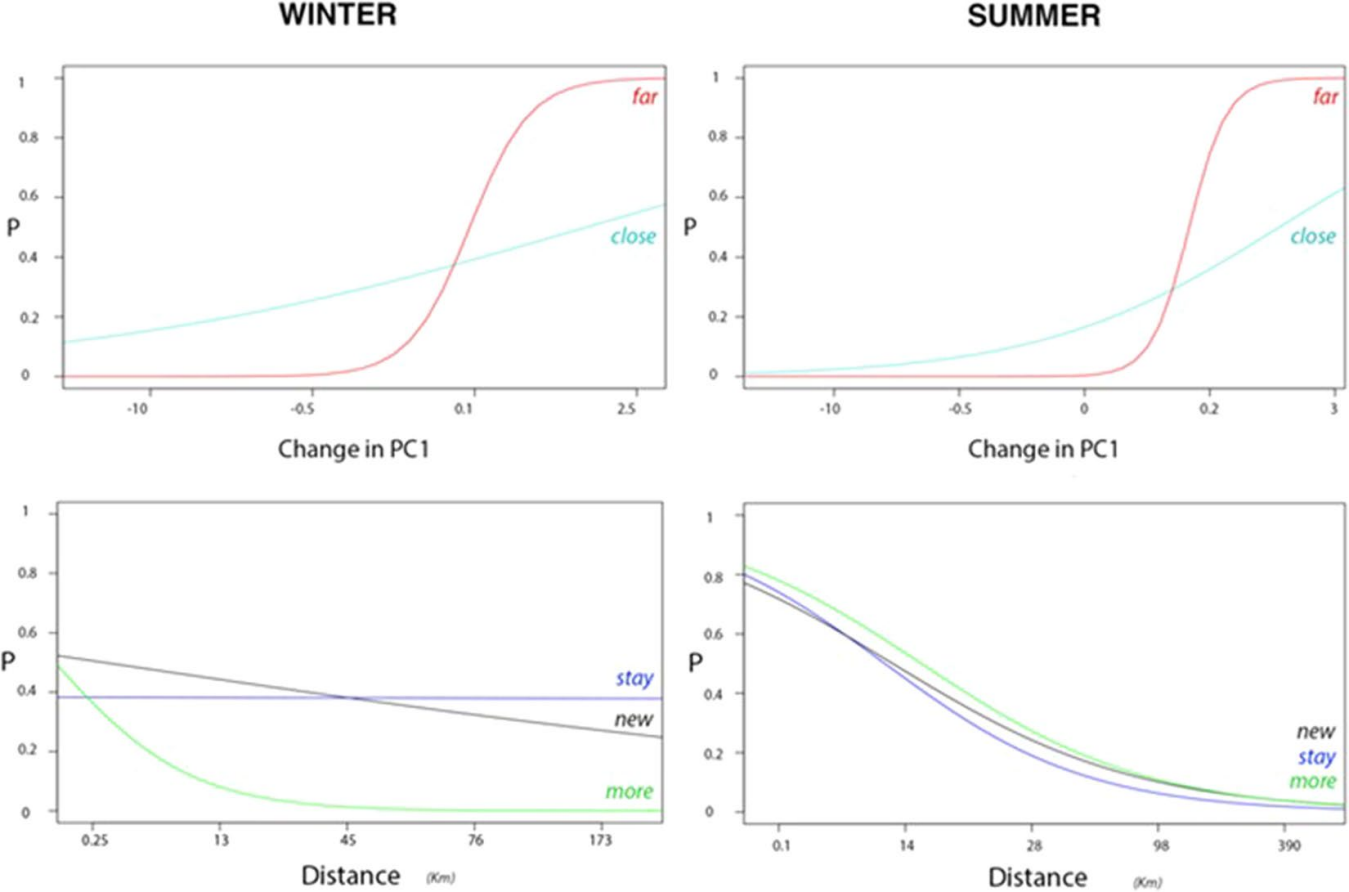
Graphical representation of the probability that GPS-marked vultures select 1 × 1 km cells in relation to the characteristics of the rewilding processes in the two study seasons. Above: Probability of vulture presence in relation to change (1956 vs 2011) in the coordinates of Principal Component 1, which describes a gradient from humanised (−) to natural habitats (+). We show separately cells “far” and “close” to the breeding area site (considering the median of the distance as the cut point). Below: Probability of use of those cells currently having “natural” habitats (above the PC1 median in 2011) in relation to the distance to the breeding area. We distinguish three categories in relation to the habitat transition observed since 1956: “*New natural*” cells rewilded recently, having passed from humanised to natural habitats; “*Stayed natural*” cells with no or low levels of rewilding; “*More natural*” cells that were natural in 1956 and have experienced rewilding.

Consistent results were found between the two seasons. Vultures were more likely to be found in natural grid cells with higher values of variation in PC1, indicating changes from humanised to natural. This effect was more marked far from the breeding area (Table 3, Fig. 3a,b) perhaps because the “far” category also includes a number of non-humanised foraging areas where rewilding processes are intense. The probability of visiting cells with negative values (cells being humanised) was always low.

**Table 3:** Selected GLMMs (lowest AICc) describing rewilding habitat use by GPS-marked griffon vultures.

Effect	Estimate	S.E.	d.f.
**Analysis 1**			
**Winter**			
Intercept	−0.4355	0.13120	29
Distance (far)	0.6002	0.02265	46147
Change in PC1	0.1266	0.01212	46147
Change in PC1*Distance (far)	0.9504	0.02572	46147
**Summer**			
Intercept	−0.5764	0.11390	26
Distance (far)	1.6681	0.02542	44147
Change in PC1	0.2083	0.01416	44147
Change in PC1*Distance (far)	1.1173	0.02952	44147
**Analysis 2**			
**Winter**			
Intercept	−0.479326	0.159003	29
Antiquity (more)	−0.078471	0.074955	14539
Antiquity (new)	0.501008	0.046633	14539
Distance	−0.003958	0.33531	14539
Distance*Antiquity (more)	−1.867788	0.099720	14539
Distance*Antiquity (new)	−0.248927	0.048505	14539
**Summer**			
Intercept	0.93271	0.13597	26
Antiquity (more)	0.33146	0.04177	22081
Antiquity (new)	0.11299	0.04959	22081
Distance	−1.03632	0.03112	22081
Distance*Antiquity (more)	−0.08515	0.4374	22081
Distance*Antiquity (new)	−0.21050	0.05699	22081

Analysis 1: probability of use in relation to the distance to the breeding area and changes in the coordinates of Principal Component 1 (that describes a gradient from humanised (−) to natural habitats (+)). Analysis 2: probability of use of cells with current natural habitats (above the PC1 median in 2011) in relation to the transition observed since 1956. In each case, the two study seasons were analysed separately.

The second analysis concerning the stages of rewilding revealed that the presence of vultures in winter was more likely in grid-cells categorised as: “*New natural*” and “*Stayed natural*”, almost independently of the distance to the breeding area. However, “*More natural*” cells were only used very close to the breeding area (Fig. 3c). During summer, the pattern of change in probabilities was almost similar for the three categories, with higher values close to the breeding area (Fig. 3d). The abundance of hunted wild ungulates was clearly higher in “*Stayed natural*”, but the abundance of livestock was similar and low in all three categories (Fig. S6).

## Discussion

Our results show that griffon vultures concentrate an important part of their foraging activities in rewilding areas where carcasses of wild ungulates have become the main trophic resource available. This provides a broader view of the role of large avian scavengers in the changing scenario of Mediterranean ecosystems and challenges the traditional perception of European vultures as almost exclusively dependent on agro-grazing activities in humanised landscapes, with few contributions of wild ungulates only in high-mountain biomes^16,18^.

Why do vultures in southern Spain concentrate an important part of their foraging behaviour in cells with natural vegetation? The abundance of wild ungulates is high in broad areas of the Sierra Morena mountains. The carrying capacity of wild ungulates has been estimated at 10 to 15 ind/km^2^ in natural areas^24^, with the number of culled individuals oscillating between 4 to 5 ind/km^2^ in Sierra Morena^25^. Moreover, at many private game properties and thanks to supplementary feeding, much higher densities of wild ungulates can be found (>30 culled individuals per km^2^)^26^. During winter, hunting activities increase the availability of wild ungulate carcasses, as a permissive regulation (Order 2th May 2012) allows the remains of the culled ungulates (guts, skins, distal part of the legs, heads of animals lacking trophy value) to be left in place to be consumed by vultures^27–29^. Moreover, some wild ungulates may escape wounded from hunting raids and die later. Accordingly, birds showed a significant preference for natural habitats (shrub followed by Mediterranean woodlands) where the densities of the preferred wild herbivores (especially red deer) reach maximum values^30^.

In summer, the hunting ban means that wild ungulate carcasses are less available, and thus vultures diversify their foraging strategies by visiting other habitats and relying more on domestic ungulates (mainly sheep), whose densities reach higher numbers in this period (15 to 20 ind/km^2^)^23^. Additionally, livestock mortality in Mediterranean extensive grazing areas occurs mainly in the summer, thus increasing carrion availability from this source. Although vultures still preferred natural habitats in the surroundings of the colony, they showed a greater propensity for open areas during this season, probably those where extensive cattle concentrate. Indeed, grasslands became more important in summer, and birds performed long-distance trips to humanised areas (more than 300 km away) where livestock is the most important food resource.

The summer season is a period of high-energy requirements because of chick rearing, but winter can also be a very demanding season because vultures need to accumulate fat reserves for the breeding period^31^. Moreover, birds have to maximise the time for foraging in winter due to the lower availability of daylight hours^32^. Accordingly, GPS-based data revealed that foraging activities lasted 3 h in winter and 6 h on average in summer. Further, vultures rely on thermal updrafts for soaring flights^33,34^, which are less frequent in winter. These dissimilarities probably change the time and energy trade-offs faced by vultures between seasons. Thus, vultures would forage mainly in the surroundings of the breeding area during winter^35^ because the high availability of food provided by hunting activities allow them to maximise food-intake rates while not incurring the high cost of locomotion imposed by adverse flight conditions. Conversely, in summer, with less availability of wild ungulate carcasses, vultures seem to show a more opportunistic foraging behaviour likely favoured by better atmospheric conditions, minimising energy expenditure during long-range movements.

### Habitat use and rewilding

In winter, population and individual scales yielded similar results regarding habitat selection, which would be explained by the fact that the vultures consistently exploited smaller and homogeneous areas during this season. In summer, however, the vultures preferentially exploited grasslands at the population scale, but a preference for scrublands reappears at the individual scale. This would be in accordance with the mixed strategy of exploitation of wild and domestic animals mentioned above, but also suggests that, even within a relatively humanised matrix occupied by agro-livestock farms, individuals actively select scrub patches. Accordingly, and especially in winter, griffon vultures used rewilding areas associated with high densities of wild ungulates^36,37^. Previous studies have shown that land abandonment is associated with the expansion of wild ungulates to the detriment of livestock and crops^38^. Hence, wild ungulates could slowly substitute livestock and sustain landscape heterogeneity^39^. Importantly, this would imply that farmers, as principal beneficiaries of the regulating services provided by scavengers, would be progressively replaced by hunters, which poses new scenarios from a conservation point of view as changes in stakeholders would lead to modifications in interest, roles and attitudes^40^. In any case, rewilding processes will determine adjustments in the carrying capacity of Mediterranean biomes for wild ungulates, so scavenger populations could also be affected^16^.

In winter, vultures seem to prefer early stages of rewilding (scrubs and scrubs with scattered trees) with relative independence of the distance to the colony, while advanced stages (with dense tree formations, characterised by higher values of change in PC1) were only used in the surroundings of the breeding area. Aspects related to the ease of finding ungulate carcasses could determine this trade-off between “naturalness” and distance to the breeding area, as it would be easier for vultures to locate and consume carcasses in areas with scrubs as compared to woodlands and dense vegetation^20^. Moreover, game hunting occurs preferentially in patches with scrubland, probably because of the greater ease of hunting with packs of dogs and shooting fleeing ungulates^41^. The same reasons could explain why our GPS-marked vultures did not prefer to forage in those places with natural habitats that had experienced more advanced rewilding stages (*More natural*) during the winter period (when wild ungulate carcasses seem to constitute the bulk of the diet). Conversely, vultures used cells with less advanced stages of rewilding, either for being in the first steps of natural succession (*New natural*)^42,43^ or for not having changed much since the 1950s (*Stay natural*). In summer, the probability of vulture presence shows a correlation with the distance again (more probable at areas closer to the breeding area) but all three stages of rewilding are used equally. Arguably, as mentioned above, this is explained by the fact that vultures respond to the lower abundance of food resources by adopting less selective and more opportunistic foraging strategies.

### Perspectives

In our study area, as in much of the mountains of southern Europe, land abandonment processes are increasing^44^, while numbers of extensive livestock (sheep and goats) are declining progressively. This has led to passive rewilding and natural succession in large areas formerly occupied by crops and pastures, which are invaded by Mediterranean scrub, while many areas already occupied by scrubs in the past have become mature forests in the present^45^. These processes have been accompanied by the recolonisation of these areas by wild ungulates^46^. Our results showed that griffon vultures respond positively to rewilding processes by focussing their foraging activities for a great part of the year in areas where re-growing natural vegetation and populations of wild ungulates predominate. What implications do these scenarios have from an ecological and conservation point of view? First, the expansion of wild ungulates within Mediterranean woodland ecosystems may turn their carcasses into natural random resources if natural deaths occur or remains are left in an unpredictable way in space and time. In this case, it would benefit the diversity and functioning of complex scavenger guilds^47^, leading to the facilitation of carcass availability for other vultures/scavengers. On the other hand, increases in wild ungulate populations and subsequent hunting activities also have consequences. Red deer and other species have often been artificially favoured by introductions and supplementary feeding for sport game purposes, in an active rewilding process^48^. During the hunting season of 2014/2015, a total number of 116,892 wild ungulates were hunted in our study region, which, in comparison to the 2003/2004 hunting season, indicates an increase of 64% and 47% in the number of red deer and wild boar (information provided by Junta de Andalucía). The process has been so pronounced that in some areas deer densities reach 50 ind/km^2^ ^49^, which may lead to overgrazing problems and hamper the ecological succession process^50,51^. In addition, the dependence on game activities will provoke the accumulation of hunting discards at predictable sites in space and/or time, leading to the monopolisation of carcasses by griffon vultures. This is known to disrupt the interspecific relationships to the detriment of less competitive avian scavenger species, as occurs in supplementary feeding stations^15,52^. Another negative effect of an increase in the consumption of game activity discards is the ingestion of lead bullets, a worldwide concern for vultures^52,53^, also detected in our study area^54^.

Land abandonment and natural succession, in concert with the increase in wild ungulate densities, are processes expected to continue in the future^10,37^, especially if the Common Agricultural Policies are liberalised^55^. Conversely, the increase of land devoted to traditional productive systems, such as the dehesas, may reverse in the future due to both abandonment of marginal zones and overgrazing in the most accessible areas^56,57^. These facts may favour an ecological dissociation between those areas subject to rewilding and other areas where agriculture and grazing intensifies, and where the trophic resources for scavenger birds are reduced or made less accessible due to the application of sanitary directives^39,58^. Consequently, it will be necessary to delve into the functioning of scavenger guilds as it confronts the dichotomy represented by wild and domestic ungulate carcasses that have a very different availability in space and time^17^, as well as detectability and accessibility. At a broader scale, in a changing scenario modelled by both ecological succession and policy decisions^56^, the functionality of scavenging processes and their actors can be largely dependent on the management of large regions subject to rewilding processes.

## Methods

### Study area

Griffon vultures were tagged in Andalusia, southern Iberia. This area encompasses 42,000 km^2^ ranging from western Sierra Morena to eastern Sierra de Cazorla, both of which are mountain ranges that embrace the Guadalquivir Valley (Fig. 1). Twelve percent of the Iberian griffon vulture population breeds in our study area^59^. The vegetation of S. Morena is dominated by large extensions of the so-called “dehesas”, which are extensive agro-pastoral ecosystems dominated by a mixture of scattered oak trees (*Quercus*. *ilex*, *Q*. *suber*, *Q*. *faginea*), grassland, cereal crops and Mediterranean scrubs^30,60^. During the last half of the 20^th^ century, natural woodlands and scrublands in S. Morena have been replaced by conifer, mainly (*Pinus pinea*) and eucalyptus (*Eucalyptus* spp.*)* plantations. S. Cazorla which is more rough and dominated by Mediterranean oak trees and pinewoods (*P*. *pinaster*, *P*. *halepensis)*^60,61^. Both S. Morena and S. Cazorla mountain ranges have a very low human population density (<3 ind/km^2^) in contrast to the Guadalquivir Valley (80 ind/km^2^), which is largely deforested and occupied by dry farming and irrigated land with high human densities. Extensive and semi-intensive livestock exploitations of sheep *(Ovis aries*), goats (*Capra hircus*) and, to a lesser extent, pigs (*Sus scrofa domestica*), horses (*Equus ferus caballus*) and cattle (*Bos taurus*), graze across the study area, with the highest densities in certain parts of S. Morena (Supporting Information, Fig. S5). Big game hunting activities (mostly red deer (*Cervus elaphus*) and wild boar (*S*. *scrofa*) take place throughout the mountain ranges but are more frequent in Sierra Morena than in Cazorla.

### GPS tracking

In December 2014, we captured and tagged 30 adult griffon vultures (information about our focal species can be found in Appendix S1 in Supporting Information) by means of cannon-nets in baited locations within the breeding area of the Cazorla Natural Park (Fig. S1). Each bird was fitted with a 98 g GPS/GPRS-GSM device with an accelerometer from e-Obs digital telemetry (http://www.e-obs.de)^62^. The devices were attached to the birds using backpack harnesses, which represent 1.1% of the weight of the captured birds (mean = 8,679 g, SD = 567). We used different device configurations to optimise energy performance across seasons (Table S1).

We split the data into two seasonal periods: fall-winter, hereafter “winter” (October 2014–February 2015) and spring-summer, “summer” (May–July 2015). We were interested in comparing the two periods because they encompass the main big-game hunting season and the closed season, respectively. The first period also overlaps with the non-breeding season (October–December) and the onset of incubation (January–February). Conversely, the summer period was of interest because there is no big-game hunting and vultures were involved in chick-rearing tasks. We decided to exclude data from March and April because during these months there was still some hunting activity and the vultures are incubating or have very small chicks. Thus, we focussed on two well-defined periods with little chance of overlap in environmental conditions and food resource availability (determined by big-game hunting).

Given that we were interested in habitat use during foraging activities, we limited locations to those that met the following characteristics: (1) Speed <0.5 m/s, which indicated that birds had landed and were possibly eating. This threshold is lower than that considered by other studies^35^ (<4 m/s), as we detected events of flying birds at very low speeds; and (2) Positions within the central hours of the day (winter: 9am–3pm UTC; summer: 8am–4pm UTC), when the availability of thermal updrafts is higher, favouring foraging activities^19,63^. Additionally, (3) all locations within the breeding area (Fig. S1) were excluded, to prevent possible biases linked to reproductive and social activities.

### Land uses and characterisation of trophic resources

Within the entire area exploited by the vultures (Fig. 1), we focussed our analyses of land-use changes and availability of trophic resources on the Andalusian region. For this area, we have good information about variables evaluating long-term changes in land use that are unavailable for neighbouring regions. This procedure restricted the analyses to 74% of the GPS locations obtained in winter and 46% in summer.

Within this study area, habitat was characterised in 41,813 1 × 1 km grid-cells by means of ArcMap 10.3^64^. Information on historical and current land uses was obtained from the databases of the Andalusian government (CORINE, SIOSE platforms; Appendix S2). We focussed our research on the two extreme years (1956 and 2011) with availability of comparable information. The original land-use categories (N = 113 in 1956 and N = 152 in 2011) were simplified by combining them into 19 final categories according to their importance and frequency of appearance (Table S2). In addition, we performed a single Principal Component Analysis merging data from the two periods. Thus, each cell-grid had one value for each year, 1956 and 2011, representing the percentage of occupancy of each land use (except “others”) allowing us to reduce the variance to a few, independent and orthogonal axes (Fig. S2; Table S3).

Following^65^, the relative abundance of wild ungulates was estimated from hunting statistics. We used a layer of hunting estates covering the whole study area and reports on the number of wild ungulates hunted from 2004 to 2008 (Andalusian government, unpublished data). We calculated a total index of hunted ungulates for each hunting estate (no. of hunted ungulates/surface area of the hunting estate). The final index of abundance for each 1 × 1 km grid-cell was calculated on the basis of the summation of indexes calculated for every hunting estate existing in the grid-cell. This index was the product of the total number of ungulates shot and the relative surface area occupied by the hunting estate within the grid-cell. The main game species were red deer (61%) and wild boar (31%). The remaining 8% included roe deer (*Capreolus capreolus*) and fallow deer (*Dama dama*). The number of wild ungulates hunted is a good proxy of wild ungulate density^66–68^. Although it is recognised that biases may exist in relation to hunting effort (e.g. the number of hunters), this problem would mainly affect rare or locally distributed game species, the relationship being valid for the most abundant taxa^69^. In our case, it should be taken into account that the index mainly reflects the abundance of two very abundant species, the red deer and wild boar, so we are confident that it is adequate.

The relative abundance of domestic livestock was calculated from data provided by the “Spanish Ministry of Agriculture” (MAGRAMA, http://www.magrama.gob.es/), which included livestock statistics of farms in each Spanish municipality. We only worked with data from those farms labelled as “extensive” because carcasses from intensive holdings are rarely available to vultures. The final index of abundance for each 1 × 1 km grid-cell was calculated on the basis of the abundance of livestock (the sum of heads of bovine, porcine, equine and sheep and goats)^18^ obtained for each municipality and the surface occupied by it within each grid-cell, following the same procedure described for wild ungulates (Fig. S5). We know that this index is highly correlated (Spearman’s r = 0.7; own data) with extensive livestock biomass availability as was estimated by Morales Reyes *et al*.^70^, so it is an adequate proxy of food availability provided by extensive grazing activities.

### Analytical procedures

#### Foraging habitat use

Firstly, to determine land use preferences of foraging vultures, we performed compositional analyses considering two operational scales^71^: one encompassing the population home range (kernel 95% of all individuals together) and the other encompassing individual home ranges (kernel 95% of each individual vulture^72^). To fit the requirements of the compositional analysis, we grouped the initial 19 habitat categories into seven new levels: dehesa, farming, grassland, Mediterranean forest, reforestation, scrubs and others (Table S2). The home range estimations were made with the help of the extension Home Range Tool for ArcMap 10.2^73^.

Secondly, we determined the influence of land use, trophic resource abundance and distance to the breeding area, on the probability of selection of 1 × 1 km cells during foraging events. We performed Generalised Linear Mixed Models (GLMM) separately for each season. The response variable was presence/absence of vultures in each cell (binomial error and logit link function). A total number of 667 and 1,187 cells with the presence of vultures in winter and summer were obtained. We then generated a similar number of pseudo-absences randomly distributed within individual home ranges (kernel 95%). Five predictors were considered: i) and ii) the scores of the cell in the PC1 and PC2 in 2011 (*Score PC1; Score PC2*), iii) the distance (*Distance*) to the breeding area in Cazorla and iv) and v) the indexes of abundance of wild ungulates (*Wild ungulates*) and livestock (*Livestock*). Given that the distance to the breeding area plays a significant role in vultures’ habitat use^74^, we also tested two-way interactions between this predictor and all the others. In all models, we considered the individual as the random term to account for the non-independence of data. We also tested whether the models improved after incorporating different correlation structures to account for spatial autocorrelation when necessary (more information in Supplementary Information, including model selection, in Appendix S3).

#### Species response to rewilding

We fitted two GLMMs (one for each period), where the response variable was the presence/absence of vultures in 1 × 1 km grid-cells (binomial distribution, logit link-function). Two predictors were fitted: i) the change between the values of the cell between 1956 and 2011 (*Change in PC1*); and ii) the distance (*Distance*) from the cell to the breeding area categorised into two levels (above and below the median value; *close* and *far*). The interaction between these two variables was also fitted. Individual identity was considered as a random term, and spatial autocorrelation effects were considered. The autocorrelation structure only improved the models corresponding to the winter period. Change in PC1 was calculated as the difference in position in the axes of a grid-cell between the years (in absolute terms). In this way, we see how much it moved through the axes and the direction of the change (to the positive part vs. negative part of the axes).

We then performed an analysis to determine what *degree of rewilding* the vultures used. For this purpose, we only used cells that had values above the median in PC1 in 2011, corresponding to natural Mediterranean habitats (woodlands and dehesas). Within these cells, we established three categories depending on the degree of rewilding: (i) “*New natural*” were those cells that had been rewilded recently, having changed from low PC1-values (below the median) in 1956, to values above the median in 2011. (ii) “*Stayed natural*” cells without or with low levels of rewilding, which means that they were natural in 1956 (above the median of PC1) and continued being natural in 2011 with similar values of change in PC1. Finally, (iii) “*More natural*” were those grid-cells that were natural in 1956 (above the median of PC1), and that ¡ had gained more positive values of change in 2011. Additionally, we fitted the distance of each cell to the breeding area (*Distance*) and the interaction between these variables. Models accounting for spatial autocorrelation were always worse than those not incorporating any spatial correlation structure in these analyses.

Competing models were selected using the small-sample-size corrected version of the Akaike information criterion (AICc). These procedures were done in R^75^ (see Appendix S3 for more details on the analytical procedures and model selection Tables S4–S5).

### Ethic statements

Capture banding and tagging of Griffon vultures were conducted under permits and following the protocols approved by the competent Regional Governments of Junta de Andalucía (AC: 30/01/2013/94), in accordance with the approved guidelines.

## Supplementary information

Supplementary information
