# Supplementary material for: Rewilding processes shape the use of Mediterranean landscapes by an avian top scavenger
Source: Sci Rep. 2020 Feb 18;10:2853. doi: 10.1038/s41598-020-59591-2 (PMC7028937; doi:10.1038/s41598-020-59591-2)
Supplement: Supplementary file 1 — Supplementary information [file 41598_2020_59591_MOESM1_ESM.docx]

Supplementary Material:

**Rewilding processes shape the use of Mediterranean landscapes by an avian top scavenger**

**P. Martin-Díaz ^a†^, A. Cortés-Avizanda ^a,b, *,†^, D. Serrano ^a^, E. Arrondo^a^, J.A. Sánchez-Zapata^c^ and J.A. Donázar ^a^**

^a^ Department of Conservation Biology, Estación Biológica Doñana (CSIC), Seville, Spain

^b^ Animal Ecology and Demography Unit, IMEDEA (CSIC-UIB),

Balearic Islands (Mallorca), Spain

**^c^** Department of applied Biology, University Miguel Hernández,

Elche (Alicante), Spain

^*^ Corresponding author: [cortesavizanda@gmail.com](mailto:cortesavizanda@gmail.com) Tel.: (+34) 971611714

ORCID NUMBER: 0000-0002-9674-6434

**^†^**Both authors contribute equally to the ms

ORCID Numbers DS: 0000-0001-5383-3944; JAD: 0000-0002-9433-9755

This material includes:

**Appendix S1**. Focal Species.

**Appendix S2**. GIS, habitat and food resource description and characterisation.

**Appendix S3**. Analytical procedures.

**Table S1:** Battery configuration of GPS devices.

**Table S2:** Original land use categories and further re-categorisation.

**Table S3:** Component loadings of the habitats of the study area.

**Table S4:** Top-ranked candidate GLMMs for habitat use in winter and summer.

**Table S5:** All candidate GLMMs for both analyses of rewilding.

**Figure S1:** Map of the study area and locations of breeding areas and nests.

**Figure S2:** Plot of the loadings corresponding to the two first Principal Components.

**Figure S3**: Spatial distribution of land-use categories in 1956-2011.

**Figure S4:** Changes in the frequencies of habitat categories between 1956 and 2011.

**Figure S5:** Spatial distribution of indexes of abundance of wild ungulates and livestock.

**Figure S6.** Food resource availability in relation to rewilding processes.

**Appendix S1. Focal species**

The griffon vulture is a long-lived and large-sized (up to 12 kg) avian scavenger species living in mountains and dry biomes of southern Europe, North Africa, the Middle East and central Asia^1^. Globally, it is considered as “Least-Concern”^2^ but European populations are now almost restricted to the Iberian Peninsula (95% of the population^3^), where the population has recovered over the last few decades and is estimated at 31,000-37,000 breeding pairs^4^. It breeds colonially in cliffs where a single chick is raised^5^. It relies on social mechanisms to locate carcasses of medium- and large-sized mammals^6,7^, mainly livestock carcasses derived from traditional agro-grazing systems^8^ but also the remains of wild ungulates^9,10^. Because of their abundance and large body size, griffon vultures are considered the main avian scavenger providing regulatory ecosystem services in Southern Europe ^11^.

The area of the Cazorla Natural Park represents an isolated breeding zone for griffon vultures within the distribution of this species in the south of the Iberian Peninsula (Figure S1). Within this area, vulture density is variable, with a maximum of breeding pairs in the central zone of the Park. In our study, the GPS-tagged vultures were distributed mainly in cliffs of these denser breeding areas (Figure S1).

**Appendix S2. GIS, habitat and food resource description and characterisation**

Geographical data (grid-cell definition, land uses, vulture locations, ungulate and livestock index as well as home ranges) were managed with ArcMap 10.3 ^12^. The coordinate system used was UTRS 1989 UTM Zone 30 (Projection= Transverse Mercaptor). Most of the analysis was performed in R software^13^.

We used the Corine layer, which has information on land uses since the middle of the 20th century, available in the database of the Andalusian government ^14^. This includes information from the following years: 1956, 1999, 2003, 2007. For the newest land-use information, we worked with the SIOSE platform ^15^, which had the layer for 2011. We focussed our research on land uses of the two extreme years of the periods available (1956 vs. 2011). For comparing the two years, we re-categorised the land-uses (N=113 in 1956 and N=152 in 2011) into a final 19 categories (Table S1). The last category “others” was excluded from the analysis, given that it was hardly represented in our study area.

In 1956, the most represented category was farming with 44% of the grid-cells of the study area (N=41813). This was followed by scrubs (34%), dehesa (13%) and Mediterranean forest (4%). In 2011, farming was again dominant (44%) followed by dehesa (22%), scrubs (20%) and Mediterranean forest (4%). The most evident changes between 1956 and 2011 are related to the growth of dehesas, the increase in scrubs (both for oaks and conifers) and reforestation in the Sierra Morena surroundings. In the Guadalquivir valley, olive groves and urban areas expanded the most, with woody crops also increasing (Fig. S2). A representation of the changes relative to each habitat category (Fig. S3) shows clear processes of rewilding. Thus, scrublands and grasslands have changed towards habitats that are more complex as dense scrublands with tree cover and woodlands. The dehesa show a particular trend. Most of the dehesa land has remained unchanged or has experienced rewilding but a portion of the other habitats, such as scrublands and grasslands, have changed to dehesas probably because of the growth of trees, as was already described for some parts of the study area ^16^. Whether the future of these dehesas is natural regeneration or a degradation to intensive grazing or agricultural use is uncertain and probably depends on the quality of the areas from an economic point of view. Thus, dehesas in marginal areas will likely be abandoned^16^.

**Appendix S3 Analytical procedures**

We used the packages *stats*^13^ and *factoextra*^17^ in Rstudio^18^. For GLMMs, we used the function glmmer in the package *lme4*^19^. We calculated all possible models, their AICc, ΔAICc, R^2^, etc. using the *MuMIn* package^20^ in R (see Table S4-S6).

**Spatial autocorrelation**

Spatial autocorrelation models were carried out in SAS 9.2 ^21^ with the GLIMMIX function and the Laplace method. Spatial autocorrelation was modeled by incorporating different correlation structures to the within-group random term (individual ID, see below). We ran a model that did not account for spatial autocorrelation and others incorporating different spatial correlation structures: Exponential, Spherical, Power, Linear Log and Gaussian. For model selection, we used the Akaike's Information Criterion corrected for small sample sizes (AICc), which defines the most parsimonious models as those with the lowest values. For some analysed periods, the model with the lowest AICc did not include the spatial autocorrelation term, but in others the best-positioned models incorporated some type of correlation structure.

**Model fitting and selection**

Generalised Linear Mixed Models^22^ were fitted in all the analytical procedures. Individual was fitted as a random term. The models accounted for spatial autocorrelation when necessary (analysis for habitat use in summer and rewilding analysis 1 for summer and winter). Apart from the predictor variables accounting for habitat characteristics and food abundance, we fitted the interaction between distance and these variables as the resources could be differentially selected in relation to the distance to the breeding area ^23^. Model selection was done using the Akaike Information Criterion corrected for small sample sizes (AICc)^24, 25^. We considered as statistically equivalent those models with Delta AICc < 2.0. All analyses were carried out in R statistical software^13^. Tables S4 and S5 show the list of candidate models.

**Table S1**

GPS device settings and time elapsed between consecutive locations. The devices were activated one hour before sunrise and turned off one hour after sunset.

|  |  | **Battery status** | | | |
| --- | --- | --- | --- | --- | --- |
|  |  | Full | Non-full | Close to security level | Under security level |
| **Griffon vulture** | **Low-performance setting** | 10 min | 30 min | 1 h | 1 day |
|  | **High-performance setting** | 5 min | 20 min | 30 min | 1 day |

**Table S2.** Original land use categories (1956 and 2011) and further re-categorisation

| **Re-categorisation (compositional analysis)** | **Re-categorisation for the remaining analyses** | **Raw Categories 1956** | **Raw Categories 2011** |
| --- | --- | --- | --- |
| **Dehesa** | Dehesa | Arable crop with tree formations: dense oaks | Arable crop with tree formations: dense oaks |
|  |  | Arable crop with tree formations: scattered oaks | Arable crop with tree formations: scattered oaks |
|  |  | Grassland with tree formations: dense conifers | Grassland with tree formations: dense conifers |
|  |  | Grassland with tree formations: scattered conifers | Grassland with tree formations: scattered conifers |
|  |  | Grassland with tree formations: conifers + eucalyptus | Grassland with tree formations: conifers + eucalyptus |
|  |  | Grassland with tree formations: eucalyptus | Grassland with tree formations: eucalyptus |
|  |  | Grassland with tree formations: other hardwood-forest species | Grassland with tree formations: other hardwood-forest species |
|  |  | Grassland with tree formations: other mixtures | Grassland with tree formations: other mixtures |
|  |  | Grassland with tree formations: dense oaks | Grassland with tree formations: dense oaks |
|  |  | Grassland with tree formations: scattered oaks | Grassland with tree formations: scattered oaks |
|  |  | Grassland with tree formations: oaks + conifers | Grassland with tree formations: oaks + conifers |
|  |  | Grassland with tree formations: oaks+ eucalyptus | Grassland with tree formations: oaks+ eucalyptus |
| **Farming** | Dry farming | Arable crops in dry land | Arable crop that is not a paddy field |
|  |  | Arable and woody crops in dry land |  |
|  |  | Arable crops and pastures |  |
|  |  | Arable crops and natural woody vegetation |  |
|  |  | Other dry framing crops |  |
|  |  | Other mosaics of crops and natural vegetation |  |
|  | Intensive farming | Irrigated woody crop: citrus | Areas without vegetation because of ploughing |
|  |  |  | Citrus |
|  |  |  | Citrus-vineyards |
|  |  |  | Intensive farming |
|  |  |  | Stone fruit - citrus |
|  | Olive grove | Woody crops in dry farming: olive grove | Fruit trees with peel-olive grove |
|  |  | Irrigated woody crop: olive grove | Stone fruits-olive grove |
|  |  | Abandoned olive groves | Fruits with seeds-olive grove |
|  |  | Olive grove-vineyard | Olive grove |
|  |  |  | Olive grove-citrus |
|  |  |  | Olive grove-vineyard |
|  | Woody crop | Woody crops in dry farming: vineyards | Fruit trees with peel |
|  |  | Other associations and mosaics of woody crops in dry land | Fruit trees with peel-stone fruit |
|  |  | Other irrigated woody crops | Other woody crops |
|  |  | Other dry faming woody crops | Stone fruits |
|  |  | Dry farming Woody crops: vineyards | Stone fruit-fruits with seeds |
|  |  | Arable crops and natural woody vegetation | Vineyard |
|  |  | Woody crops and pastures |  |
|  |  | Other abandoned woody crops |  |
|  | Irrigated land | Irrigated arable crops: not irrigated | Rafts of irrigation and livestock |
|  |  | Irrigated arable crops: irrigated and not irrigated |  |
|  |  | Irrigated arable and woody crops |  |
|  |  | Irrigated arable and woody crops: not irrigated |  |
|  |  | Irrigated arable and woody crops: Partially irrigated |  |
|  |  | Irrigated woody crops: partially or not irrigated |  |
|  |  | Mosaic of irrigated woody crops |  |
|  |  | Mosaic of dry farming and irrigated arable crops |  |
|  |  | Mosaic of dry farming and irrigated arable and woody crops |  |
|  |  | Mosaic of dry farming and irrigated woody crops |  |
|  |  | Other irrigated arable crops |  |
| **Grassland** | Grassland | Areas that suffered erosive processes | Areas that suffered erosive processes |
|  |  | Grassland with clearings | Firebreaks |
|  |  | Continuous grassland | Grassland with clearings |
|  |  | Crags and bare soil | Continuous grassland |
|  |  |  | Cleared soil |
| **Mediterranean forest** | Mediterranean forest | Woody crops and natural woody vegetation | Dense tree formations: other hardwood-forest species |
|  |  | Dense tree formations: other hardwood-forest species | Dense tree formations: other mixtures |
|  |  | Dense tree formations: other mixtures | Dense tree formations: oaks |
|  |  | Dense tree formations: oaks | Dense tree formations: oaks and conifers |
|  |  | Dense tree formations: oaks and conifers | Dense tree formations: oaks and eucalyptus |
|  |  | Dense tree formations: oaks and eucalyptus |  |
| **Scrubs** | Dense scrubs with conifers and eucalyptus | Dense scrubs with tree formations: dense conifers | Dense scrubs with tree formations: dense conifers |
|  |  | Dense scrubs with tree formations: scattered conifers | Dense scrubs with tree formations: scattered conifers |
|  |  | Dense scrubs with tree formations: conifers + eucalyptus | Dense scrubs with tree formations: conifers + eucalyptus |
|  |  | Dense scrubs with tree formations: eucalyptus | Dense scrubs with tree formations: eucalyptus |
|  |  | Dense scrubs with tree formations: oaks + conifers | Dense scrubs with tree formations: oaks + conifers |
|  |  | Dense scrubs with tree formations: oaks + eucalyptus | Dense scrubs with tree formations: oaks + eucalyptus |
|  | Dense scrubs with oaks and other hardwood-forest species | Dense scrubs with tree formations: other hardwood-forest species | Dense scrubs with tree formations: other hardwood-forest species |
|  |  | Dense scrubs with tree formations: other mixtures | Dense scrubs with tree formations: other mixtures |
|  |  | Dense scrubs with tree formations: dense oaks | Dense scrubs with tree formations: dense oaks |
|  |  | Dense scrubs with tree formations: scattered oaks | Dense scrubs with tree formations: scattered oaks |
|  | Scattered scrubs with conifers and eucalyptus | Scattered scrubs with tree formations: dense conifers | Scattered scrubs with tree formations: dense conifers |
|  |  | Scattered scrubs with tree formations: scattered conifers | Scattered scrubs with tree formations: scattered conifers |
|  |  | Scattered scrubs with tree formations: conifers + eucalyptus | Scattered scrubs with tree formations: conifers + eucalyptus |
|  |  | Scattered scrubs with tree formations: eucalyptus | Scattered scrubs with tree formations: eucalyptus |
|  |  | Scattered scrubs with tree formations: oaks + conifers | Scattered scrubs with tree formations: oaks + conifers |
|  |  | Scattered scrubs with tree formations: oaks + eucalyptus | Scattered scrubs with tree formations: oaks+ eucalyptus |
|  | Scattered scrubs with oak and other hardwood-forest species | Scattered scrubs with tree formations: other hardwood-forest species | Scattered scrubs with tree formations: other hardwood-forest species |
|  |  | Scattered scrubs with tree formations: other mixtures | Scattered scrubs with tree formations: other mixtures |
|  |  | Scattered scrubs with tree formations: dense oaks | Scattered scrubs with tree formations: dense oaks |
|  |  | Scattered scrubs with tree formations: scattered oaks | Scattered scrubs with tree formations: scattered oaks |
|  | Scattered scrubs with pastures | Scattered scrubs with pastures | Scattered scrubs with pastures |
|  |  | Scattered scrubs with grassland and rocks/soil | Scattered scrubs with grassland and rocks/soil |
|  | Scrubs | Dense scrubs | Dense scrubs |
|  |  | Other abandoned woody crops |  |
| **Reforestation** | Reforestation | Dense tree formations: conifers | Dense tree formations: conifers |
|  |  | Dense tree formations: conifers + eucalyptus | Dense tree formations: conifers + eucalyptus |
|  |  | Dense tree formations: eucalyptus | Dense tree formations: eucalyptus |
|  |  | Loggings and recent forest plantations |  |
|  |  | Burned spaces | Burned spaces |
|  |  | Grassland with tree formations: dense conifers |  |
| **Others** | Rivers | Rivers and channels: gallery forest | Channeled rivers |
|  |  | Rivers and channels: sheet of water | Rivers and channels: and other riparian shapes |
|  |  | Rivers and channels: and other riparian shapes | Rivers and channels: gallery forest |
|  |  |  | Watercourses |
|  |  |  | Natural water flows: water sheets |
|  | Roads | Highways and roads | Rail network |
|  |  |  | Road network |
|  |  |  | Not paved communications paths |
|  |  |  | Pedestrian zones, packings, roads |
|  | Urban area | Airports | Airports |
|  |  | Paddy field | Agrarian-residential areas |
|  |  | Sports and recreations equipment | Agrarian-farming areas |
|  |  | Other technical infrastructures | Paddy field |
|  |  | Urban area | Industrial/mining rafts |
|  |  | Agrarian and residential urbanisations | Camping |
|  |  | Urbanisations and residential buildings | Golf links |
|  |  | Construction spaces | Urban centre |
|  |  | Industrial and commercial spaces | Cemetery |
|  |  | Urban green spaces | Circuits for races and training |
|  |  | Mining spaces | Commercial and offices |
|  |  | Port facilities | Administrative institutional complex |
|  |  | Areas without vegetation because of ploughing | Commercial and recreational complex |
|  |  | No category assigned | Cultural complex |
|  |  | Water irrigation pod | Educational complex |
|  |  | Industrial or mine raft | Hotel resorts |
|  |  |  | Industrial complex |
|  |  |  | Religious complex |
|  |  |  | Sanitary complex |
|  |  |  | Vegetable water deposits |
|  |  |  | Wastewater treatment and water purification plants |
|  |  |  | Junkyards |
|  |  |  | Widening |
|  |  |  | Rubbish dumps |
|  |  |  | Isolated industries |
|  |  |  | Technical infrastructures |
|  |  |  | Forestry installations |
|  |  |  | Gas pipeline/oil pipeline |
|  |  |  | Basketball facilities |
|  |  |  | Water pipelines |
|  |  |  | Football facilities |
|  |  |  | Paddle tennis facilities |
|  |  |  | Tennis facilities |
|  |  |  | Wind power facilities (Eolic-facilities) |
|  |  |  | Electrical installations |
|  |  |  | Hydroelectric facilities |
|  |  |  | Military installations |
|  |  |  | Solar installations |
|  |  |  | Other sport facilities |
|  |  |  | Recreation park |
|  |  |  | Technological/business park |
|  |  |  | Parks |
|  |  |  | Parks, squares, gardens, seaside promenades |
|  |  |  | Penitentiary |
|  |  |  | Treatment plants |
|  |  |  | Bullfighting arenas |
|  |  |  | Industrial estate (organised) |
|  |  |  | Industrial estate (unorganised) |
|  |  |  | Sports centres |
|  |  |  | Coastal protection |
|  |  |  | Yachting port |
|  |  |  | Fairground |
|  |  |  | Discharges extractions |
|  |  |  | Green spaces with gardens |
|  |  |  | Service stations |
|  | No category assigned | Artificial channels | Channel without vegetation |
|  |  | Railway complex | Water reservoir (dams) |
|  |  | Indoor growing (under plastic) | Temporal greenhouses |
|  |  | Dams: foil water | Permanent greenhouses |
|  |  | Tailings and waste dumps | Artificial water sheet |
|  |  | Estuaries and tidal channels | Lakes and lagoons |
|  |  | Continental lagoons | Marisma with vegetation |
|  |  | Coastline lagoons | Marisma without vegetation |
|  |  | Seas and oceans | Archaeological site |
|  |  | Tidal marshland with vegetation | Lagoon vegetation |
|  |  | Marisma with vegetation (not tidal) | beaches, dunes and sandbanks |
|  |  | Recent marsh without vegetation | Gibraltar |
|  |  | Beaches, dunes and sandbanks | Heliport |
|  |  | Industrial salt mines and gardens with crops | Racecourse |
|  |  | Traditional salt mines | Industrial salt flats and gardens with crops |
|  |  | River channel without vegetation | Traditional salt flats |
|  |  | Nuclear installations | Nuclear installations |
|  |  |  | Thermic installations |
|  |  |  | Telecommunications |
|  |  |  | Tropical Fruits |

**Table S3.** Factor loadings after Principal Component Analysis for the 18 categories assigned to land uses in the study area (see Table S2). The 19^th^ category “others” was excluded because it was not representative enough.

| **Land uses** | **PC1** | **PC2** |
| --- | --- | --- |
| Mediterranean forest | 0.157 | -0.205 |
| Intensive farming | -0.069 | 0.027 |
| Woody crop | -0.096 | -0.022 |
| Dry farming | -0.477 | 0.046 |
| Dehesa | 0.222 | -0.250 |
| Dense scrubs with conifers | 0.147 | 0.554 |
| Dense scrubs with oaks | 0.359 | -0.260 |
| Scattered scrubs with conifers | 0.175 | 0.440 |
| Scattered scrubs with pastures | 0.231 | 0.162 |
| Scattered scrubs with oaks | 0.386 | -0.432 |
| Scrubs | 0.244 | 0.011 |
| Olive grove | -0.374 | -0.010 |
| Grassland | 0.198 | 0.067 |
| Reforestation | 0.152 | 0.684 |
| Irrigated land | -0.156 | 0.046 |
| Rivers | -0.134 | 0.055 |
| Roads | -0.304 | 0.035 |
| Urban area | -0.401 | 0.024 |
|  |  |  |
| Eigenvalue | 1.96 | 1.63 |
| Variance (%) | 10.3 | 8.6 |

**Table S4.** Top-ranked candidate GLMMs for the analysis of habitat use during the winter and summer periods. The selected model is the first model (in bold and italic) in each period. It was selected on the basis of the AICc values. The AICc value, the difference in AICc with respect to the highest-ranked model (ΔAICc), the AICc weight, the residual degrees of freedom (df), the maximised value of the log-likelihood function (logLik), and the coefficients of determination (R^2^ and _adj_R^2^) are shown. Coefficients of determination were calculated with the function r.squaredGLMM() of the package *MuMIn* of R.

| **Period** | **AIC** | **ΔAIC** | **Weight** | **Model** | **df** | **logLik** | **R2** | **adjR2** |
| --- | --- | --- | --- | --- | --- | --- | --- | --- |
| **Winter** |  |  |  |  |  |  |  |  |
|  | ***55628.81*** | ***0*** | ***0.97*** | ***PC1+*** | ***11*** | ***-27803.4*** | ***0.17*** | ***0.22*** |
|  |  |  |  | ***PC2+*** |  |  |  |  |
|  |  |  |  | ***Wild ungulates +*** |  |  |  |  |
|  |  |  |  | ***Livestock+*** |  |  |  |  |
|  |  |  |  | ***Distance +*** |  |  |  |  |
|  |  |  |  | ***Distance*PC1 +*** |  |  |  |  |
|  |  |  |  | ***Distance*PC2 +*** |  |  |  |  |
|  |  |  |  | ***Distance*Wild ungulates +*** |  |  |  |  |
|  |  |  |  | ***Distance*Livestock*** |  |  |  |  |
|  | 55635.59 | 6.78 | 0.03 | PC1+ | 10 | -27807.79 | 0.17 | 0.22 |
|  |  |  |  | PC2+ |  |  |  |  |
|  |  |  |  | Wild ungulates + |  |  |  |  |
|  |  |  |  | Livestock+ |  |  |  |  |
|  |  |  |  | Distance + |  |  |  |  |
|  |  |  |  | Distance*PC1 + |  |  |  |  |
|  |  |  |  | Distance*PC2 + |  |  |  |  |
|  |  |  |  | Distance*Livestock |  |  |  |  |
|  | 55682.39 | 53.58 | 0 | PC1+ | 10 | -27831.19 | 0.17 | 0.22 |
|  |  |  |  | PC2+ |  |  |  |  |
|  |  |  |  | Wild ungulates + |  |  |  |  |
|  |  |  |  | Livestock+ |  |  |  |  |
|  |  |  |  | Distance+ |  |  |  |  |
|  |  |  |  | Distance*PC1+ |  |  |  |  |
|  |  |  |  | Distance*PC2+ |  |  |  |  |
|  |  |  |  | Distance*Wild ungulates |  |  |  |  |
|  | 55686.90 | 58.09 | 0 | PC1+ | 9 | -27834.45 | 0.17 | 0.22 |
|  |  |  |  | PC2+ |  |  |  |  |
|  |  |  |  | Wild ungulates + |  |  |  |  |
|  |  |  |  | Livestock+ |  |  |  |  |
|  |  |  |  | Distance + |  |  |  |  |
|  |  |  |  | Distance*PC1+ |  |  |  |  |
|  |  |  |  | Distance*PC2 |  |  |  |  |
|  | 55688.20 | 59.39 | 0 | PC1+ | 10 | -27834.1 | 0.17 | 0.22 |
|  |  |  |  | PC2+ |  |  |  |  |
|  |  |  |  | Wild ungulates + |  |  |  |  |
|  |  |  |  | Livestock+ |  |  |  |  |
|  |  |  |  | Distance+ |  |  |  |  |
|  |  |  |  | Distance*PC1 + |  |  |  |  |
|  |  |  |  | Distance*Wild ungulates |  |  |  |  |
|  |  |  |  | Distance*Livestock |  |  |  |  |
|  | 55692.39 | 63.58 | 0 | PC1+ | 9 | -27837.19 | 0.17 | 0.22 |
|  |  |  |  | PC2+ |  |  |  |  |
|  |  |  |  | Wild ungulates + |  |  |  |  |
|  |  |  |  | Livestock+ |  |  |  |  |
|  |  |  |  | Distance + |  |  |  |  |
|  |  |  |  | Distance*PC1 + |  |  |  |  |
|  |  |  |  | Distance*Livestock |  |  |  |  |
|  | 55718.92 | 90.11 | 0 | PC1+ | 8 | -27851.46 | 0.17 | 0.22 |
|  |  |  |  | Wild ungulates + |  |  |  |  |
|  |  |  |  | Livestock+ |  |  |  |  |
|  |  |  |  | Distance + |  |  |  |  |
|  |  |  |  | Distance*PC1 + |  |  |  |  |
|  |  |  |  | Distance*Livestock |  |  |  |  |
|  | 55719.69 | 90.88 | 0 | PC1+ | 9 | -27856.83 | 0.17 | 0.22 |
|  |  |  |  | Wild ungulates + |  |  |  |  |
|  |  |  |  | Livestock+ |  |  |  |  |
|  |  |  |  | Distance + |  |  |  |  |
|  |  |  |  | Distance*PC1 + |  |  |  |  |
|  |  |  |  | Distance*Wild ungulates+ |  |  |  |  |
|  |  |  |  | Distance*Livestock |  |  |  |  |
|  | 55731.67 | 102.86 | 0 | PC1+ | 9 | -27856.83 | 0.17 | 0.22 |
|  |  |  |  | PC1+ |  |  |  |  |
|  |  |  |  | PC2+ |  |  |  |  |
|  |  |  |  | Wild ungulates + |  |  |  |  |
|  |  |  |  | Livestock+ |  |  |  |  |
|  |  |  |  | Distance+ |  |  |  |  |
|  |  |  |  | Distance*PC1 |  |  |  |  |
|  |  |  |  | Distance*Wild ungulates |  |  |  |  |
|  | 55734.32 | 105.51 | 0 | PC1+ | 8 | -27859.16 | 0.17 | 0.22 |
|  |  |  |  | PC2+ |  |  |  |  |
|  |  |  |  | Wild ungulates + |  |  |  |  |
|  |  |  |  | Livestock |  |  |  |  |
| **Summer** |  |  |  |  |  |  |  |  |
|  | ***40065.81*** | ***0*** | ***1*** | ***PC1+*** | ***11*** | ***-20021.9*** | ***0.32*** | ***0.42*** |
|  |  |  |  | ***PC2+*** |  |  |  |  |
|  |  |  |  | ***Wild ungulates+*** |  |  |  |  |
|  |  |  |  | ***Livestock+*** |  |  |  |  |
|  |  |  |  | ***Distance +*** |  |  |  |  |
|  |  |  |  | ***Distance*PC1+*** |  |  |  |  |
|  |  |  |  | ***Distance*PC2+*** |  |  |  |  |
|  |  |  |  | ***Distance*Wild ungulates+*** |  |  |  |  |
|  |  |  |  | ***Distance*Livestock*** |  |  |  |  |
|  | 40097.07 | 31.26 | 0 | PC1+ | 10 | -20038.53 | 0.32 | 0.42 |
|  |  |  |  | PC2+ |  |  |  |  |
|  |  |  |  | Wild ungulates+ |  |  |  |  |
|  |  |  |  | Livestock+ |  |  |  |  |
|  |  |  |  | Distance+ |  |  |  |  |
|  |  |  |  | Distance*PC1+ |  |  |  |  |
|  |  |  |  | Distance*Wild ungulates+ |  |  |  |  |
|  |  |  |  | Distance*Livestock |  |  |  |  |
|  | 40119.06 | 53.25 | 0 | PC1+ | 10 | -20049.53 | 0.32 | 0.42 |
|  |  |  |  | PC2+ |  |  |  |  |
|  |  |  |  | Wild ungulates+ |  |  |  |  |
|  |  |  |  | Livestock+ |  |  |  |  |
|  |  |  |  | Distance+ |  |  |  |  |
|  |  |  |  | Distance*PC1+ |  |  |  |  |
|  |  |  |  | Distance*PC2+ |  |  |  |  |
|  |  |  |  | Distance*Livestock |  |  |  |  |
|  | 40120.74 | 54.93 | 0 | PC1+ | 10 | -20050.37 | 0.32 | 0.42 |
|  |  |  |  | PC2+ |  |  |  |  |
|  |  |  |  | Wild ungulates+ |  |  |  |  |
|  |  |  |  | Livestock+ |  |  |  |  |
|  |  |  |  | Distance+ |  |  |  |  |
|  |  |  |  | Distance*PC1+ |  |  |  |  |
|  |  |  |  | Distance*PC2+ |  |  |  |  |
|  |  |  |  | Distance*Wild ungulates |  |  |  |  |
|  | 40156.23 | 90.42 | 0 | PC1+ | 9 | -20069.11 | 0.32 | 0.42 |
|  |  |  |  | PC2+ |  |  |  |  |
|  |  |  |  | Wild ungulates+ |  |  |  |  |
|  |  |  |  | Livestock+ |  |  |  |  |
|  |  |  |  | Distance+ |  |  |  |  |
|  |  |  |  | Distance*PC1 |  |  |  |  |
|  |  |  |  | Distance*Livestock |  |  |  |  |
|  | 40158.41 | 92.6 | 0 | PC1+ | 9 | -20070.2 | 0.32 | 0.42 |
|  |  |  |  | Wild ungulates+ |  |  |  |  |
|  |  |  |  | Livestock+ |  |  |  |  |
|  |  |  |  | Distance+ |  |  |  |  |
|  |  |  |  | Distance*Wild ungulates+ |  |  |  |  |
|  |  |  |  | Distance*Livestock |  |  |  |  |
|  | 40159.16 | 93.35 | 0 | PC1+ | 9 | -20070.58 | 0.32 | 0.42 |
|  |  |  |  | PC2+ |  |  |  |  |
|  |  |  |  | Wild ungulates+ |  |  |  |  |
|  |  |  |  | Livestock+ |  |  |  |  |
|  |  |  |  | Distance*PC1+ |  |  |  |  |
|  |  |  |  | Distance*Wild ungulates+ |  |  |  |  |
|  | 40173.24 | 107.43 | 0 | PC1+ | 9 | -20077.62 | 0.32 | 0.42 |
|  |  |  |  | PC2+ |  |  |  |  |
|  |  |  |  | Wild ungulates+ |  |  |  |  |
|  |  |  |  | Livestock+ |  |  |  |  |
|  |  |  |  | Distance*PC1+ |  |  |  |  |
|  |  |  |  | Distance*PC2+ |  |  |  |  |
|  | 40218.11 | 152.3 | 0 | PC1+ | 8 | -200101.06 | 0.31 | 0.42 |
|  |  |  |  | PC2+ |  |  |  |  |
|  |  |  |  | Wild ungulates+ |  |  |  |  |
|  |  |  |  | Livestock+ |  |  |  |  |
|  |  |  |  | Distance+ |  |  |  |  |
|  |  |  |  | Distance*PC1 |  |  |  |  |
|  | 40226.72 | 160.91 | 0 | PC1+ | 8 | -20105.36 | 0.31 | 0.42 |
|  |  |  |  | Wild ungulates+ |  |  |  |  |
|  |  |  |  | Livestock+ |  |  |  |  |
|  |  |  |  | Distance+ |  |  |  |  |
|  |  |  |  | Distance*Wild ungulates |  |  |  |  |

**Table S5.** All candidate models for both GLMM analyses of rewilding. First model in bold and italic is the one selected.

| **Period** | **AIC** | **ΔAIC** | | | **Weight** | | | | | **Model** | | | | **df** | | **logLik** | | | **R^2^** | | | **_adj_R^2^** | | |  |  |
| --- | --- | --- | --- | --- | --- | --- | --- | --- | --- | --- | --- | --- | --- | --- | --- | --- | --- | --- | --- | --- | --- | --- | --- | --- | --- | --- |
| 1. ***Response to rewilding*** | | | | | | | | | | | | | | | | | | | | | | | | | | |
| *Winter* | | | | | | | |  | | | |  | | |  | | |  | | |  | | |  |  |  |
|  | ***60054.14*** | | | ***0.000*** | | | ***1*** | | | | ***Change in PC1+*** | | | | ***5*** | | ***-30022.07*** | | | ***0.082*** | | | ***0.11*** | | |  |
|  |  | | |  | | |  | | | | ***Distance+***  ***Change in PC1*Distance*** | | | |  | |  | | |  | | |  | | |  |
|  | 61570.97 | | | 1516.82 | | | 0 | | | | Distance + | | | | 4 | | -30781.48 | | | 0.052 | | | 0.07 | | |  |
|  |  | | |  | | |  | | | | Change in PC1 | | | |  | |  | | |  | | |  | | |  |
|  | 62294.14 | | | 2239.99 | | | 0 | | | | Change in PC1 | | | | 3 | | -31144.07 | | | 0.037 | | | 0.045 | | |  |
|  |  | | |  | | |  | | | |  | | | |  | |  | | |  | | |  | | |  |
|  | 63030.78 | | | 2976.63 | | | 0 | | | | Distance | | | | 3 | | -31512.39 | | | 0.021 | | | 0.028 | | |  |
|  |  | | |  | | |  | | | |  | | | |  | |  | | |  | | |  | | |  |
|  | 64023.06 | | | 3968.92 | | | 0 | | | | Intercept only | | | | 2 | | -32009.53 | | | 0 | | | 0 | | |  |
|  |  | | |  | | |  | | | |  | | | |  | |  | | |  | | |  | | |  |
|  | | | | | | | | | | | |  | | |  | | |  | | |  | | |  |  |  |
| *Summer* | | |  | | | | | |  | | | |  | |  | | |  | | |  | | |  |  |  |
|  | ***50266.98*** | ***0.000*** | | | | | | ***1*** | | | | ***Change in PC1+*** | | | ***5*** | | | ***-25128.49*** | | | ***0.21*** | | | ***0.28*** |  |  |
|  |  |  | | | | | |  | | | | ***Distance+*** | | |  | | |  | | |  | | |  |  |  |
|  |  |  | | | | | |  | | | | ***Change in PC1*Distance*** | | |  | | |  | | |  | | |  |  |  |
|  | 51852.72 | 1585.74 | | | | | | 0 | | | | Distance + | | | 4 | | | -25922.36 | | | 0.18 | | | 0.25 |  |  |
|  |  |  | | | | | |  | | | | Change in PC1 | | |  | | |  | | |  | | |  |  |  |
|  | 53982.31 | 3715.33 | | | | | | 0 | | | | Distance | | | 3 | | | -26988.16 | | | 0.14 | | | 0.19 |  |  |
|  |  |  | | | | | |  | | | |  | | |  | | |  | | |  | | |  |  |  |
|  | 56889.49 | 6622.51 | | | | | | 0 | | | | Change in PC1 | | | 3 | | | -28441.75 | | | 0.085 | | | 0.11 |  |  |
|  |  |  | | | | | |  | | | |  | | |  | | |  | | |  | | |  |  |  |
|  | 60821.69 | 10554.71 | | | | | | 0 | | | | Intercept only | | | 2 | | | -30408.85 | | | 0 | | | 0 |  |  |
|  |  |  | | | | | |  | | | |  | | |  | | |  | | |  | | |  |  |  |
|  |  |  | | |  | | | | | |  | | | |  | | |  | | |  | | |  |  |  |
| 1. ***Rewilding stages*** | | | | |  | | | | | |  | | | |  | | |  | | |  | | |  |  |  |
| *Winter* | | |  | | |  | | | | |  | | | |  | | |  | | |  | | |  |  |  |
|  | ***17258.90*** | ***0.0000*** | | | ***1*** | | | | | | ***Antiquity+*** | | | | ***7*** | | | ***-8622.445*** | | | ***0.17*** | | | ***0.23*** |  |  |
|  |  |  | | |  | | | | | | ***Distance+*** | | | |  | | |  | | |  | | |  |  |  |
|  |  |  | | |  | | | | | | ***Antiquity*Distance*** | | | |  | | |  | | |  | | |  |  |  |
|  | 17669.98 | 411.08 | | | 0 | | | | | | Distance+ | | | | 5 | | | -8829.988 | | | 0.15 | | | 0.20 |  |  |
|  |  |  | | |  | | | | | | Antiquity | | | |  | | |  | | |  | | |  |  |  |
|  | 17757.97 | 499.07 | | | 0 | | | | | | Antiquity | | | | 4 | | | -8874.983 | | | 0.14 | | | 0.19 |  |  |
|  |  |  | | |  | | | | | |  | | | |  | | |  | | |  | | |  |  |  |
|  | 17872.00 | 613.11 | | | 0 | | | | | | Distance | | | | 3 | | | -8933.002 | | | 0.14 | | | 0.18 |  |  |
|  |  |  | | |  | | | | | |  | | | |  | | |  | | |  | | |  |  |  |
|  | 18050.92 | 792.02 | | | 0 | | | | | | Intercept only | | | | 2 | | | -9023.459 | | | 0.13 | | | 0.17 |  |  |
|  |  |  | | |  | | | | | |  | | | |  | | |  | | |  | | |  |  |  |
| *Summe*r | | |  | | |  | | | | |  | | | |  | | |  | | |  | | |  |  |  |
|  | ***22644.27*** | ***0.00000*** | | | ***1*** | | | | | | ***Antiquity+*** | | | | ***7*** | | | ***-11315.13*** | | | ***0.13*** | | | ***0.19*** |  |  |
|  |  |  | | |  | | | | | | ***Distance +*** | | | |  | | |  | | |  | | |  |  |  |
|  |  |  | | |  | | | | | | ***Antiquity*Distance*** | | | |  | | |  | | |  | | |  |  |  |
|  | 22655.09 | 10.82 | | | 0 | | | | | | Distance+ | | | | 5 | | | -11322.54 | | | 0.13 | | | 0.19 |  |  |
|  |  |  | | |  | | | | | | Antiquity | | | |  | | |  | | |  | | |  |  |  |
|  | 22712.41 | 68.14 | | | 0 | | | | | | Distance | | | | 3 | | | -11353.21 | | | 0.13 | | | 0.19 |  |  |
|  |  |  | | |  | | | | | |  | | | |  | | |  | | |  | | |  |  |  |
|  | 25129.18 | 2484.91 | | | 0 | | | | | | Antiquity | | | | 4 | | | -12560.59 | | | 0.028 | | | 0.04 |  |  |
|  |  |  | | |  | | | | | |  | | | |  | | |  | | |  | | |  |  |  |
|  | 25377.81 | 2733.53 | | | 0 | | | | | | Intercept only | | | | 2 | | | -12686.90 | | | 0.017 | | | 0.02 |  |  |

**Figure S1.** Breeding distribution of griffon vultures in and around the Cazorla Natural Park. The colour of the occupied 10x10 km squares shows the number of breeding pairs in 2008 ^26^. Blue points show the position of nests occupied by GPS-tagged vultures. These nests appear outside breeding squares because after 2008 the vultures expanded to new neighbouring zones, but no reliable censuses are available. (Census from Del Moral, 2009 ^26^).


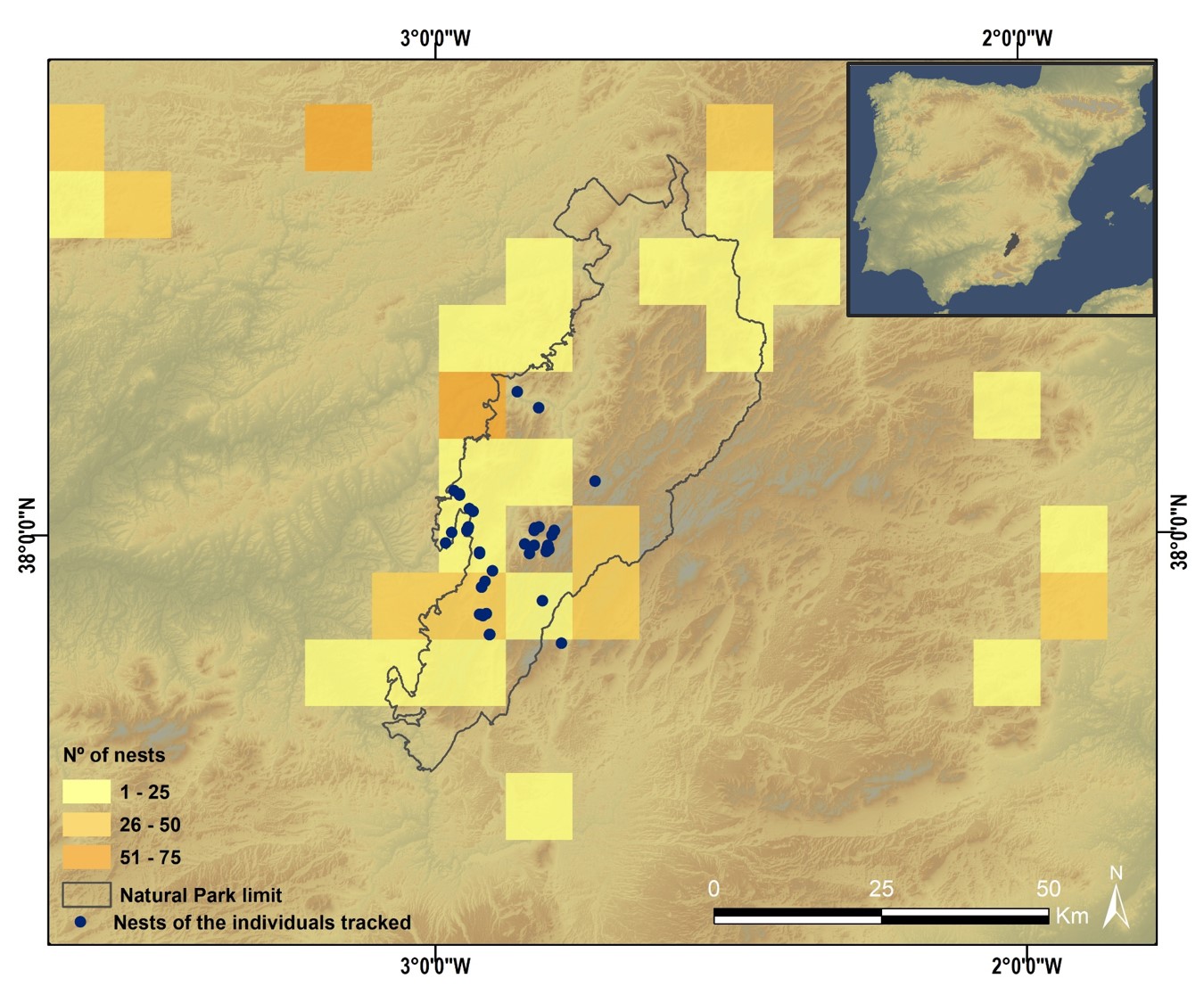

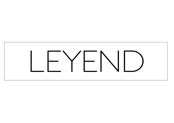


**Figure S2.** Principal Component Analysis (PCA) plot showing the loadings of the first two Principal Components. Vectors indicate the direction and strength of each land use category to the overall distribution, i.e. how they contribute in a different way to category clustering.


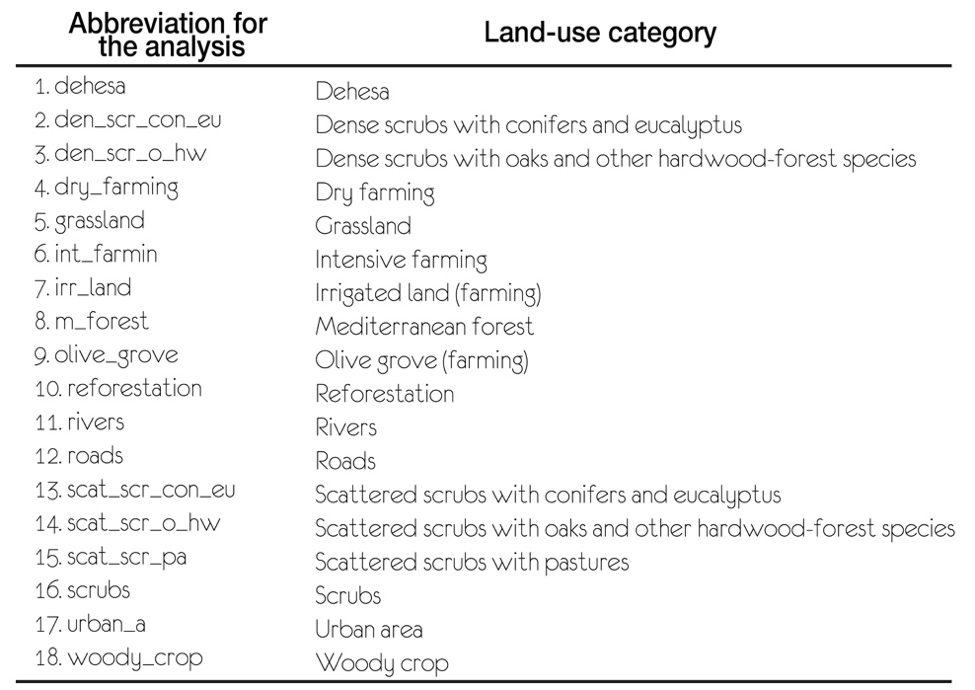

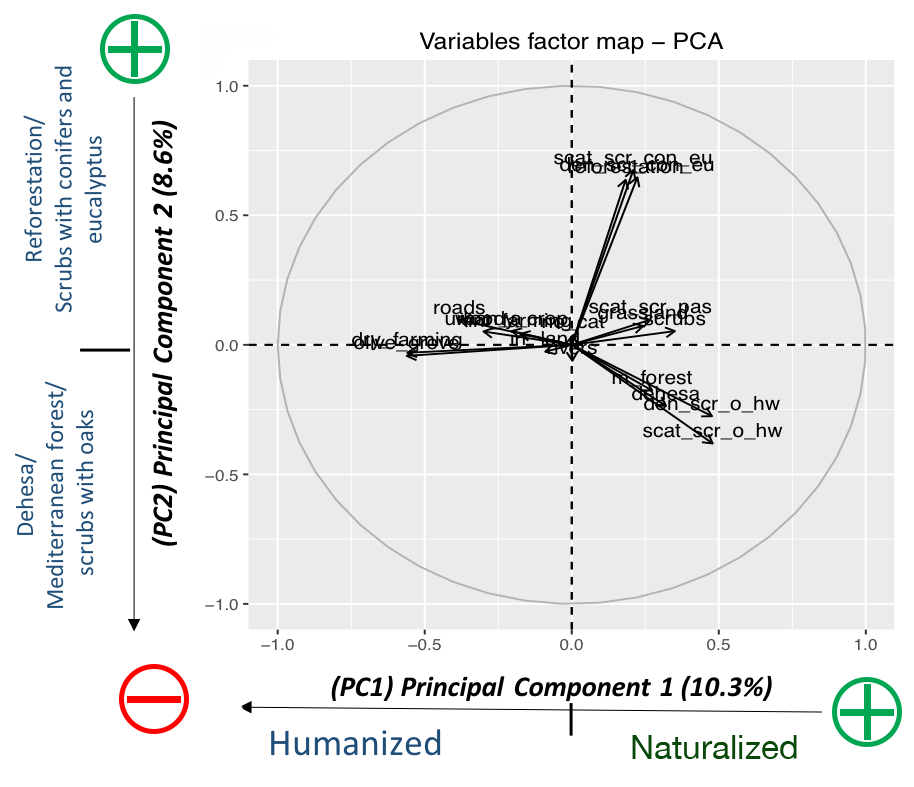


**Figure S3.** Spatial distribution of land-use categories in 1956 and 2011. **
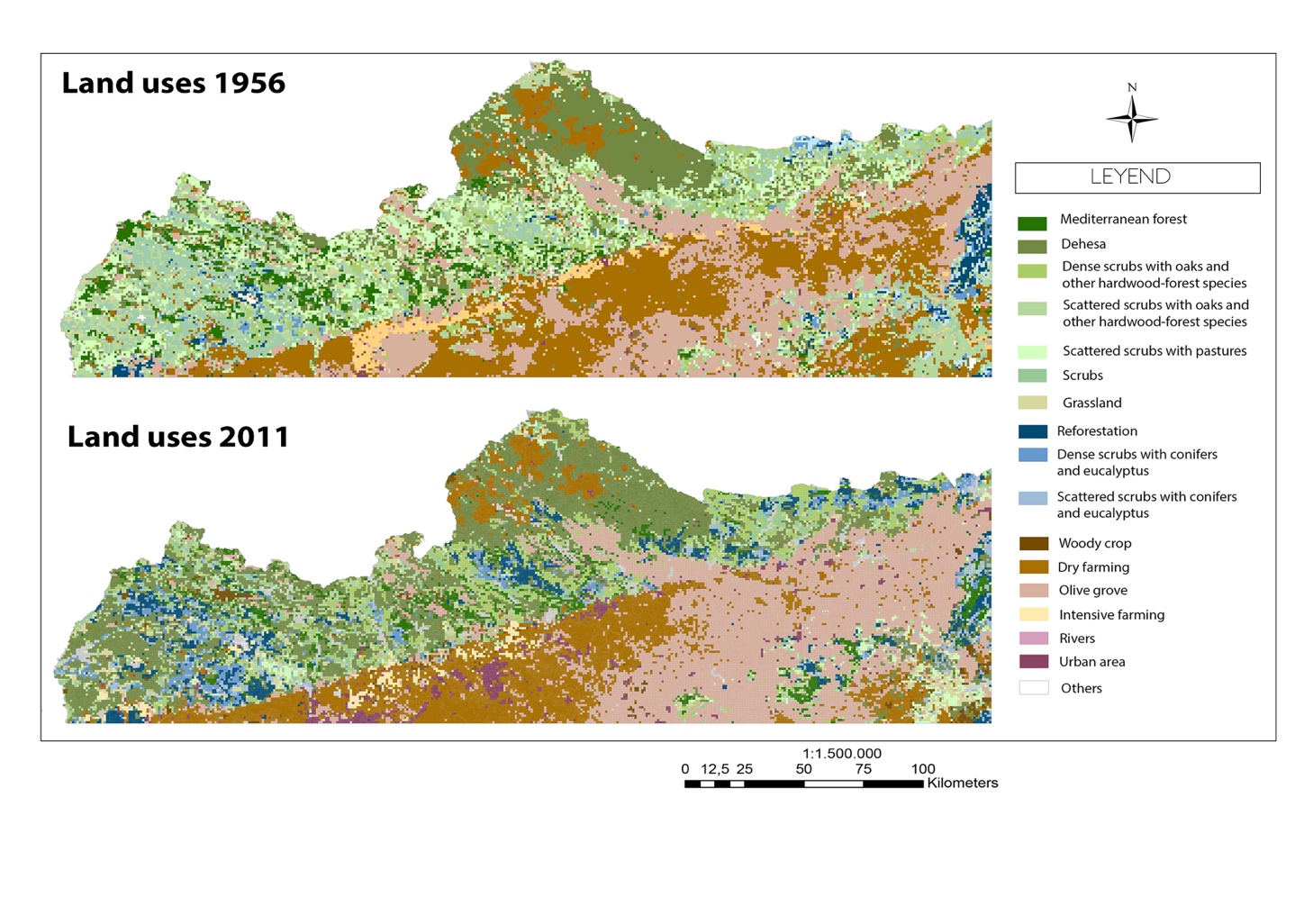
**

**Figure S4.** Changes in habitat between 1956 and 2011. For those areas occupied by each category of habitat in 1956, the distribution of frequencies of 1x1 km squares occuppied by each habitat in 2011 is shown. The black column represents the frequency of the same habitat in 2011. Note that more mature-less humanised habitats appear on the left part of the x axis.


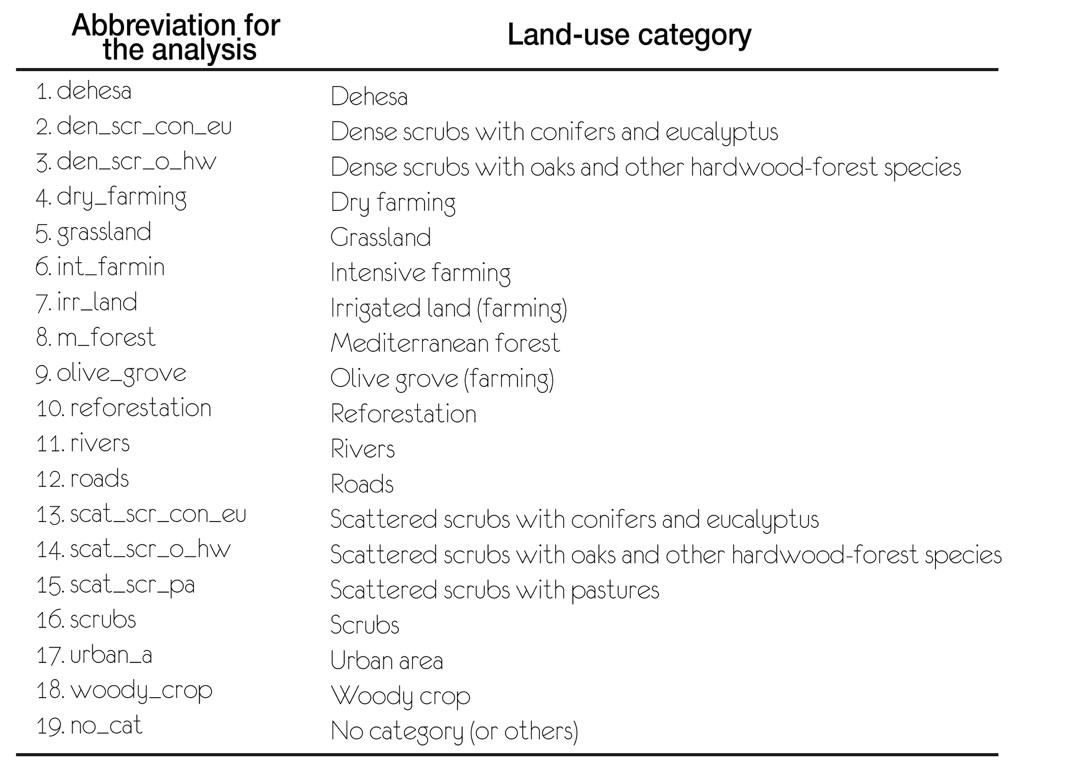


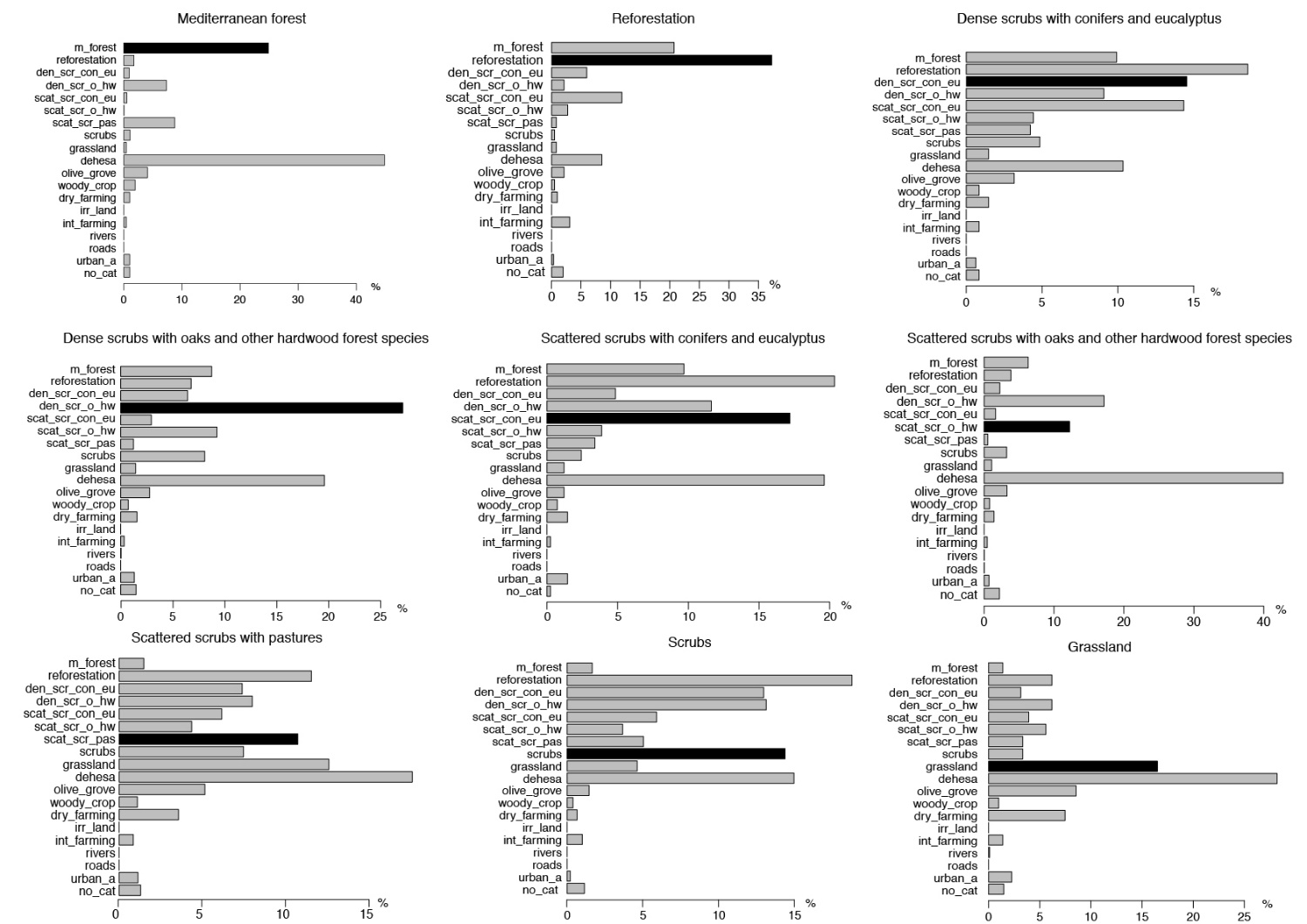


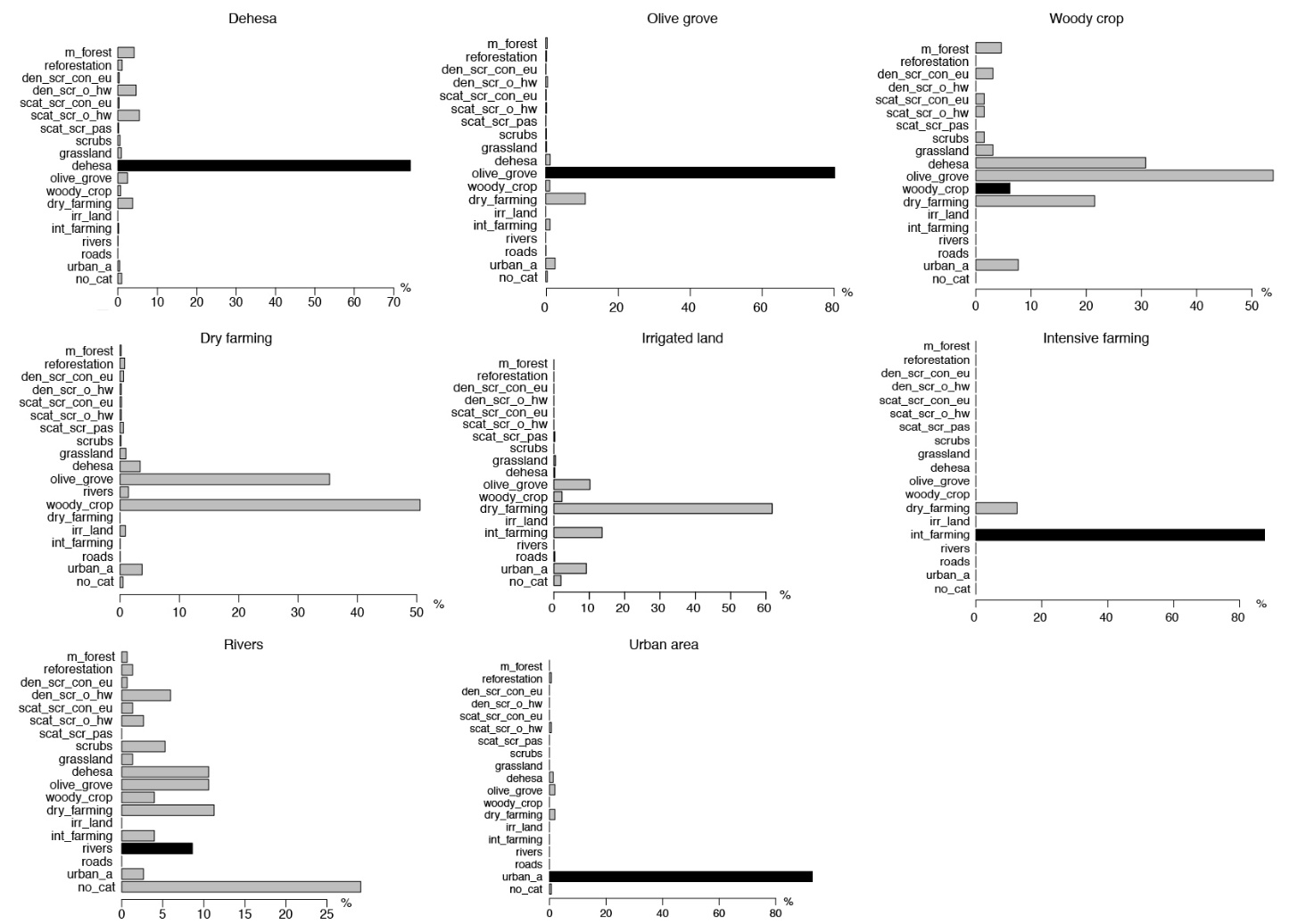


**Figure S5.** Spatial distribution of indexes of abundance of livestock and wild ungulates. The highest densities of domestic and wild ungulates were found in Sierra Morena (northern part of the study area) but they tend to occupy different portions of the mountain range.

**Figure S6** Food resource availability (wild ungulates and livestock) in relation to rewilding processes. “New natural” cells have rewilded recently, passing from humanised (1956) to natural habitats (2011); “Stayed natural” cells are those with no or low levels of rewilding; “More natural” cells were already natural in 1956 but have experienced rewilding. Results for the two study seasons are shown separately; winter (October-February), summer (May-July).


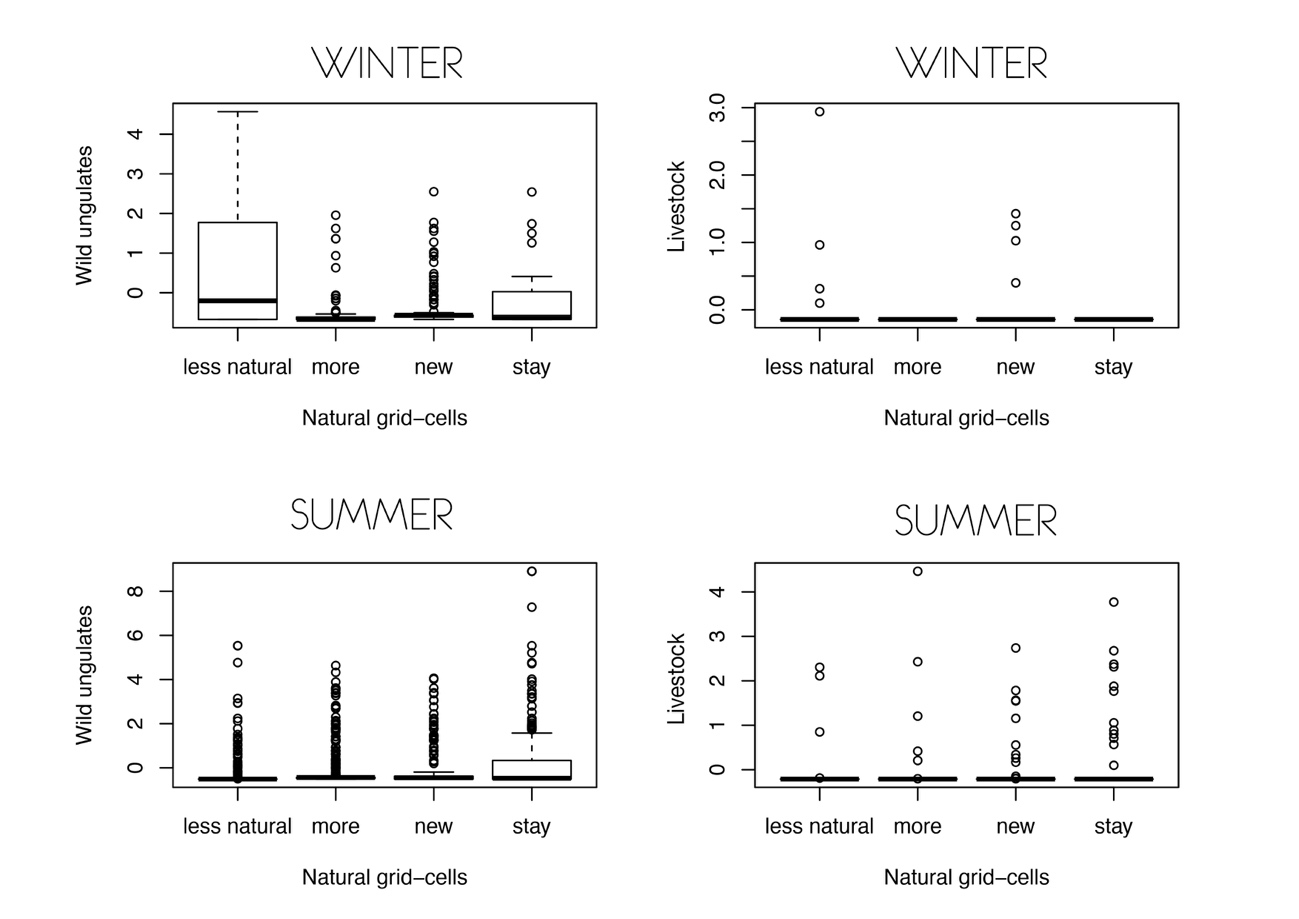


**References**

1. Slotta-Bachmayr L, Bögel R, Cardenal A (2005). The Eurasian Griffon vulture Gyps fulvus in Europe and the Mediterranean. Status report and Action Plan.

2. BirdLife International. (2018). Species factsheet: Gyps fulvus. Retrieved 8 May 2016, from http://www.birdlife.org

3. Sánchez-Zapata, J. A. (2012). Buitres y servicios ecosistemicos: investigación aplicada a la conservación de la biodiversidad y los procesos ecológicos. Eubacteria, 29, 1–2.

4. BirdLife International. (2015). European Red List of Birds. Luxembourg: Office for Official Publications of the European Communities.

5. Margalida A, Bertran J (2010). Copulatory behaviour in the colonial Eurasian Griffon vulture Gyps fulvus. Journal of Ethology, 28(1), 179–182. http://doi.org/10.1007/s10164-009-0169-5

6. Cortés-Avizanda A, Jovani R, Donázar JA, Grimm V (2014). Bird sky networks: How do avian scavengers use social information to find carrion? Ecology, 95(7), 1799–1808. http://doi.org/10.1890/13-0574.1

7. Jackson AL, Ruxton GD, Houston DC (2008). The effect of social facilitation on foraging success in vultures: a modelling study. Biology Letters, 4(3), 311–3. http://doi.org/10.1098/rsbl.2008.0038

8. Margalida A, Colomer MA (2012). Modelling the effects of sanitary policies on European vulture conservation. Scientific Reports, 2, 753. http://doi.org/10.1038/srep00753

9. Donázar JA (1993). Los buitres ibéricos: biología y conservación. (J. Reyero, Ed.). Madrid.

10. Margalida A, Colomer MA, Sanuy D (2011). Can Wild Ungulate Carcasses Provide Enough Biomass to Maintain Avian Scavenger Populations? An Empirical Assessment Using a Bio-Inspired Computational Model. PLoS ONE, 6(5), 20248. http://doi.org/10.1371/journal.pone.0020248

11. Morales-Reyes Z, Pérez-García J, Moleón M, Botella F, Carrete M, Lazcano C, et al. (2015). Supplanting ecosystem services provided by scavengers raises greenhouse gas emissions. Scientific Reports, 5, 7811. http://doi.org/10.1038/srep07811

12. ESRI. (2014). ArcGIS Desktop:ArcMap 10.3. Redlands, CA:Environmental Systems Research Institute: Esri Inc, All Rights Reserved.

13. R Core Team. (2013). R: A language and environment for statistical computering. Vienna, Austria.: R Fundation for Statistical Computing. ISBN 3-900051-07-0, http://www.R-project.org/.

14. Consejería de Medio Ambiente y Ordenación del Territorio. (2007). Mapa de usos y coberturas vegetales del suelo en Andalucía (MUCVA) 1956-1977-1984-1999-2003-2007, scale 1:25.000. Retrieved 10 January 2015, from http://www.juntadeandalucia.es/medioambiente/site/rediam/menuitem.04dc44281e5d53cf8ca78ca731525ea0/?vgnextoid=ca74d2aa40504210VgnVCM1000001325e50aRCRD&vgnextchannel=7b3ba7215670f210VgnVCM1000001325e50aRCRD&vgnextfmt=rediam

15. Junta de Andalucía (2011). Pasarela De Siose Andalucía 2011 Modelo Relacional a Un Modelo Plano (Ocupación Del Suelo). Junta de Andalucía: Consejería de Medio Ambiente y Ordenación Del Territorio.

16. Paniza A (2015). The landscape of the dehesa in the Sierra Morena of Jaén (Spain)-the transition from traditional to new land uses. Landscape Online 43:1-15. DOI 10.3097/LO.201543

17. Kassambara A, Mundt F (2017). Package ’factoextra’ : Extract and Visualize the Results of Multivariate Data Analyses. Retrieved from <http://www.sthda.com/english/rpkgs/factoextra%0ABugReports>

18. RStudio Team (2015). RStudio: Integrated Development for R. RStudio, Inc., Boston, MA URL <http://www.rstudio.com/>.

19. Bates D, Maechler M, Bolker B, Walker S, Haubo R, Christensen B, et al. (2018). Package ‘lme4’: Linear Mixed-Effects Models using ‘Eigen’ and S4. Retrieved from https://github.com/lme4/lme4/ <http://lme4.r-forge.r-project.org/>

20. Barton, K (**2013)** MuMIn: Multimodel inference. R package version 1.9.13. <http://CRAN.R-project.org/package=MuMIn>

21. SAS Institute Inc. (2011). Base SAS. Cary, NC, USA: SAS Institute Inc.: Procedures Guide.

22. Zuur AF, Ieno EN, Walker N, Saveliev AA, Smith G M (2009). Mixed effects models and extensions in ecology with R. New York, NY. http://doi.org/10.1007/978-0-387-87458-6

23. Carrete M, Donázar JA (2005). Application of central-place foraging theory shows the importance of Mediterranean dehesas for the conservation of the cinereous vulture, Aegypius monachus. Biological Conservation, 126(4), 582–590. http://doi.org/10.1016/J.BIOCON.2005.06.031

24. Burnham KP, Anderson DR (2004). Multimodel Inference Understanding AIC and BIC in Model Selection. Sociological Methods & Research, 33(2), 261–304. <http://doi.org/10.1177/0049124104268644>

25. Sugiura, N. (1978). Further analysis of the data by Akaike’s information criterion and the finite corrections. Communications in Statistics, Theory and Methods, A7, 13-26. doi:10.1080/03610927808827599

26. Del Moral JC (2009). El buitre leonado en España. Población reproductora en 2008 y métodos de censo. El buitre leonado en España. Población reproductora en 2008 y métodos de censo ((Ed.)). Madrid.
